# Perinatal Maternal Depressive Symptoms and Brain Connectivity Among 9- to 15-Year-Old Offspring

**DOI:** 10.1001/jamanetworkopen.2025.23978

**Published:** 2025-07-31

**Authors:** Dogukan Koc, Adriana P. C. Hermans, Bing Xu, Ryan L. Muetzel, Hanan El Marroun, Henning Tiemeier

**Affiliations:** 1 The Generation R Study Group, Erasmus MC, University Medical Center Rotterdam, Rotterdam, the Netherlands; 2 Department of Child and Adolescent Psychiatry/Psychology, Erasmus MC, University Medical Center Rotterdam, Rotterdam, the Netherlands; 3 Department of Radiology and Nuclear Medicine, Erasmus MC University Medical Center, Rotterdam, the Netherlands; 4 Department of Psychology, Education and Child Studies, Erasmus School of Social and Behavioral Science, Erasmus University Rotterdam, Rotterdam, the Netherlands; 5 Department of Social and Behavioral Sciences, Harvard T.H. Chan School of Public Health, Boston, Massachusetts

## Abstract

**Importance:**

Maternal depressive symptoms during the perinatal period have been associated with offspring neurodevelopment. However, the longitudinal association of prenatal and postnatal maternal depressive symptoms with the developmental change in functional brain networks among offspring remains unclear.

**Objective:**

To examine the associations of prenatal and postnatal maternal depressive symptoms with offspring functional brain connectivity development from midchildhood to adolescence.

**Design, Setting, and Participants:**

This single-site, population-based prospective cohort was embedded in the Generation R Study, conducted in Rotterdam, the Netherlands. All pregnant individuals with an expected delivery date between April 1, 2002, and January 31, 2006, were invited to participate. The study included 2825 mother-child dyads with 3627 resting-state functional magnetic resonance imaging (fMRI) scans. Participants were eligible if they had valid resting-state fMRI data from at least 1 of the 2 waves of neuroimaging. Maternal depressive symptoms were assessed during pregnancy and in the early postnatal period. Offspring underwent 2 neuroimaging assessments at age 9 to 15 years. Data were analyzed from February to December 2024.

**Exposure:**

Maternal depressive symptoms were assessed using the Brief Symptom Inventory during midpregnancy and at 2 and 6 months post partum. Depressive symptom levels were analyzed both continuously and as clinically relevant dichotomized categories.

**Main Outcomes and Measures:**

The primary outcomes were brainwide graph theory metrics, including integration (global efficiency) and segregation (modularity, clustering coefficient) derived from resting-state functional connectivity (RSFC). Secondary outcomes included within-RSFC and between-RSFC networks, defined by the Gordon parcellation.

**Results:**

The study included 2825 mother-child dyads (mean [SD] maternal age at intake, 31.1 [4.7] years; 1496 female children [53.0%]). Prenatal depressive symptoms were associated with greater increases in global efficiency (β = 0.004, SE = 0.000; false discovery rate [FDR]–corrected *P* = .002), modularity (β = 0.003, SE = 0.000; FDR-corrected *P* = .002), and within-network default mode network connectivity (β = 0.010, SE = 0.001; FDR-corrected *P* = .002) from midchildhood to adolescence. Postnatal depressive symptoms showed no significant association with offspring functional connectivity measures. Baseline internalizing problems were associated with greater increases in global efficiency among children exposed to clinically relevant prenatal depressive symptoms (β = 0.243, SE = 0.37; FDR-corrected *P* = .001).

**Conclusions and Relevance:**

In this population-based cohort study of 9- to-15-year-old children, prenatal maternal depressive symptoms were associated with differences in the trajectories of functional brain connectivity. These findings highlight the relevance of prenatal maternal mental health in understanding offspring neurodevelopmental processes.

## Key Points

**Question:**

Are prenatal and postnatal maternal depressive symptoms associated with a change in child functional brain connectivity from childhood to adolescence?

**Findings:**

In this population-based cohort study of 2825 mother-child dyads, prenatal, but not postnatal, maternal depressive symptoms were associated with a steeper increase in functional brain network integration and segregation from childhood to adolescence. Among children exposed to clinically relevant prenatal maternal depressive symptoms, more severe internalizing problems were associated with greater increases in brain network integration.

**Meaning:**

These findings suggest that prenatal maternal depressive symptoms are associated with changes in the offspring brain functional network, particularly during adolescence.

## Teaser

This cohort study examines the associations of prenatal and postnatal maternal depressive symptoms with offspring functional brain connectivity development from midchildhood to adolescence.

## Introduction

Perinatal depressive symptoms affect approximately 10% of women, imposing a significant public health burden worldwide.^1,2,3^ Exposure to perinatal maternal depressive symptoms can have persistent negative consequences on offspring, including increased risks for social-emotional problems, cognitive development, and depression.^4,5,6,7^

Functional magnetic resonance imaging (fMRI) studies have investigated the neurobiological correlates of perinatal maternal depression, frequently examining differences in resting-state functional connectivity (RSFC) in key brain regions, such as the amygdala and prefrontal cortex.^8,9^ Findings, however, remain inconsistent, likely due to methodological differences.^8,9^ For example, Qiu et al^10^ reported increased amygdala connectivity in infants exposed to prenatal depression, while Posner et al^11^ found decreased connectivity with the dorsal prefrontal cortex. Complementing these infant findings, fetal and neonatal studies have also reported altered RSFC in association with maternal distress, suggesting early in utero functional reorganization.^12,13^ However, no consistent associations have been found in early childhood.^8,9^

Longitudinal studies tracking RSFC beyond early childhood are needed to understand persistent associations between perinatal maternal depression and RSFC development.^7^ However, most prior studies relied on assessments of a single time point and narrowly defined networks. To overcome these limitations, graph theory metrics offer a network-based approach to assess brainwide functional organization.^14^ Metrics such as global efficiency, modularity, and the clustering coefficient quantify integration and segregation, capturing the brain’s capacity for distributed communication and local specialization. These measures enable a systems-level investigation of neurodevelopmental trajectories.^15,16^ In addition, prior studies rarely incorporated emotional or behavioral assessments, limiting insight into how maternal symptoms are temporally associated with later neurodevelopment and mental health difficulties among offspring.

In this population-based cohort study, we investigated how perinatal maternal depressive symptoms are associated with offspring RSFC development from midchildhood to adolescence. Building on previous work on structural connectivity,^17^ we hypothesized that prenatal depressive symptoms would be initially associated with lower global efficiency and modularity, followed by catch-up changes with increasing age. We further expected these associations to be most pronounced in networks supporting emotion regulation and attention.^18^ Finally, we hypothesized that clinically relevant perinatal maternal depressive symptoms would be associated with changes in the temporal dynamics^19,20^ of a child’s emotional symptoms and functional brain connectivity.

## Methods

### Participants

Participants were drawn from the Generation R Study, a population-based cohort study in Rotterdam, the Netherlands. All pregnant individuals with an expected delivery date between April 1, 2002, and January 31, 2006, were invited to participate. Details on recruitment were reported elsewhere.^21,22^ Written informed consent was obtained from parents and children. The Medical Ethics Committee of Erasmus Medical Centre, Rotterdam, approved the study. This study followed the Strengthening the Reporting of Observational Studies in Epidemiology (STROBE) reporting guideline.

From 8756 pregnancies, 2117 children were excluded because prenatal maternal depressive symptom data were missing, yielding 6639 children. Of these, 3387 underwent at least 1 fMRI scan. Exclusions due to excessive motion (n = 454), poor coregistration (n = 12), incidental findings (n = 24), or twin or triplet inclusion (n = 72) resulted in a final sample of 2825 mother-child pairs with 3627 scans (eFigure 2 and eTable 1 in Supplement 1). For postnatal depressive symptoms, a sample of 2260 children with available postnatal data contributed 2923 scans (eFigure 2 and eTable 2 in Supplement 1).

### Maternal Depression Symptoms

Maternal depressive symptoms were assessed during pregnancy (20-week gestation) and postnatally (at 2 and 6 months post partum) using the 6-item depression subscale of the Brief Symptom Inventory (range, 0-4, with higher scores indicating more severe depressive symptoms).^23^ In secondary analyses, we dichotomized the Brief Symptom Inventory scores based on Dutch norms,^23^ considering weighted scores above 0.75 as clinically relevant depressive symptoms. Diagnostic validity has been confirmed via the Composite International Diagnostic Interview and the Edinburgh Postnatal Depression Scale (eMethods in Supplement 1).

### Child Problem Behavior

Children reported emotional and behavioral problems via the Achenbach System of Empirically-Based Assessment.^24^ Internalizing (eg, anxiety or depression, withdrawal) and externalizing (eg, aggression, rule-breaking) scores were computed.

### Image Acquisition, Preprocessing, and Quality Control

Magnetic resonance imaging scans were acquired on a 3.0 Tesla GE Discovery MR750w system. Participants were invited to 2 neuroimaging visits: T1 and T2. Participants were instructed to lie awake in the scanner (with eyes closed) for 5 minutes and 52 seconds. No formal monitoring of wakefulness was performed.

Preprocessing was performed using fMRIPrep, version 20.1.1^25^ and included volume realignment with MCFLIRT (FSL [FMRIB Software Library]), slice-time correction with 3dTshift (AFNI [Analysis of Functional Neuroimage]), and coregistration to the corresponding T1w reference. Images were spatially normalized to the ICBM 152 Nonlinear Asymmetrical template, version 2009c^26^ by nonlinear registration with the antsRegistration tool of ANTs, version 2.1.0^27^ and resampled to Cifti format in 32k fs_LR surface space.

A combination of visual inspection and automated methods was used to assess the usability of fMRI data. To address motion-related confounders, we applied a conservative multistep correction strategy, including exclusion of high-motion scans based on framewise displacement thresholds,^28^ patient-level ICA-AROMA (Independent Component Analysis–based Automatic Removal of Motion Artifacts) denoising,^29^ and inclusion of mean framewise displacement as a covariate in models (eMethods in Supplement 1).

### Graph Theoretical Analyses and Functional Network Connectivity

For graph theory metrics and functional network connectivity analyses (eFigure 1B in Supplement 1), the brain was parcellated into 333 cortical regions of interest defined by the Gordon parcellation.^30^ Functional connectivity matrices were generated using a blood oxygen level–dependent time series.

### Graph Theory Metrics

We removed negative connections and Fisher *r*-to-*z* transformed each matrix. We generated weighted, undirected graphs to investigate brainwide functional network architecture. We characterized 3 key metrics of brainwide functional network architecture using the Brain Connectivity Toolbox in Python, version 3.9.0 (Python Software Foundation).^14^ Global efficiency assesses the mean inverse of the shortest path length, indicating how easily nodes connect through a few edges, reflecting network integration and information sharing.^31,32^ *Modularity* describes clusters of nodes (modules) with dense connections within modules and sparse connections between modules. This metric captures the ability to organize into distinct communities and reflects functional segregation.^14,31,32^ The clustering coefficient quantifies the interconnectivity among neighboring nodes and their tendency to cluster together. Both modularity and the clustering coefficient quantify the topologic segregation in brain networks, where segregation refers to the neuronal processing carried out among groups of regions or within modules.^31,32^ Higher values on these metrics indicate greater network integration, segregation, and clustering coefficients, respectively.

### Within- and Between-Network Connectivity

Pairwise Pearson correlations of the residualized regional time series were calculated across regions of interest, and the resulting connectivity estimates were Fisher *z* transformed.^30^ This study focused on 12 specific networks, as shown in eFigure 1B in Supplement 1. Network-related functional connectivity was characterized by 2 distinct measures (eFigure 4 in Supplement 1): within-network connectivity, which involves computing the mean connectivity within each network, and between-network connectivity, which entails calculating the mean connectivity values between one network and every other network.

### Covariates

Potential covariates were selected a priori based on previous research^8,9^: maternal age at prenatal intake, national origin, educational level, marital status, substance use (tobacco, cannabis, alcohol) during pregnancy, monthly household income, and child age at the MRI assessment, sex at birth, and mean framewise displacement (eMethods in Supplement 1).

### Statistical Analysis

Statistical analysis was conducted from February to December 2024. All analyses were conducted using R, version 4.3.2 (R Project for Statistical Computing).^33^ We calculated descriptive statistics for our study population (mean values of continuous variables and the proportions of categorical variables).

#### Maternal Depressive Symptoms (Continuously Modeled)

To explore the association between prenatal and postnatal maternal depressive symptoms and offspring RSFC from midchildhood to adolescence, we used a linear mixed-effects model. First, we examined the association between prenatal maternal depressive symptoms and the graph theory measures; these analyses were repeated with the postnatal maternal depressive symptoms as the exposure. The overall associations of maternal depressive symptoms over time were derived from 1 set of models (no age interaction), while the measures’ change over time (interaction: maternal depressive symptoms score × child age) was estimated in separate models. To address repeated observations within individuals, we incorporated exposure variables and covariates as fixed effects, along with a random intercept. For secondary outcomes, within- and between-network RSFC were similarly modeled.

#### Clinically Relevant Maternal Depressive Symptoms (Dichotomously Modeled)

In addition, in secondary analyses, we dichotomized the study population into those exposed to clinically relevant maternal depressive symptoms and a nonexposed reference group. We then used a generalized additive mixed model^34^ to investigate the association between exposure to clinically relevant prenatal and postnatal maternal depressive symptoms and offspring functional brain connectivity over time, aiming to capture better nonlinear changes that the linear mixed-effects model might miss (eMethods and eFigure 1C in Supplement 1).

We used a multigroup bivariate latent change score model^19^ to assess whether clinically relevant maternal depressive symptoms were associated with the temporal dynamics between child internalizing problems and functional brain connectivity (eMethods and eFigure 1C in Supplement 1). We tested baseline correlations, whether baseline brain metrics were associated with internalizing changes, and vice versa, and whether changes co-occurred over time.

All models were adjusted for child sex assigned at birth, age at neuroimaging assessment, and the aforementioned covariates. In postnatal models, prenatal maternal depressive symptoms were included to account for confounding due to symptom continuity across time. Postnatal symptoms were not included in prenatal models, as adjusting for postexposure variables may introduce bias by conditioning on potential mediators along the causal pathway.^35^ The time difference between the behavioral and resting-state fMRI assessment was adjusted in bivariate latent change score models.

#### Nonresponse and Sensitivity Analyses

We tested for selective attrition using *t* tests, Wilcoxon tests, and χ^2^ tests (eTable 4 in Supplement 1). Attrition was further addressed using inverse probability weighting (eTable 5 in Supplement 1). Sensitivity analyses included excluding offspring exposed to antidepressants in utero and testing sex interactions.

#### Imputation and Multiple Testing Correction

Missing covariates were imputed using multivariate chained equations.^36^ Multiple testing was controlled via the Benjamini-Hochberg false discovery rate (FDR) correction.^37^ Statistical significance was set at a 2-sided α < .05.

## Results

### Descriptive Information

The study included 2825 mother-child dyads (mean [SD] maternal age at intake, 31.1 [4.7] years; 1496 female children [53.0%] and 1329 male children [47.0%]) (Table 1). The mean (IQR) scores for maternal depressive symptoms during pregnancy and the postnatal period were 0.20 (IQR, 0-0.22) and 0.20 (IQR, 0-0.25), respectively. Depressive symptoms showed moderate correlations across time points (Spearman coefficients ranging from 0.47 to 0.59), with the strongest correlation between 20-week gestation and 2 months post partum. Children were, on average, 10 years of age at T1 and 14 years at T2 (eFigure 3 in Supplement 1). We present the estimated RSFC for ages 9 to 15 years; less than 10% of scans were assessed outside this age range.

**Table 1.** Descriptive Statistics of the Study Population

Characteristic	Total study population (N = 2825)^a^
**Maternal characteristics**	
Age at intake, mean (SD), y	31.1 (4.7)
National origin, No. (%)	
Dutch	1694 (60.0)
Non-Dutch European	244 (8.6)
Non-European	
African	157 (5.6)
Asian Oceanian	182 (6.4)
Caribbean	280 (9.9)
Moroccan or Turkish	268 (9.5)
Marital status: with partner	2506 (88.7)
Educational level, No. (%)	
Primary or lower	171 (6.1)
Secondary	1158 (41.0)
Higher	1496 (53.0)
Monthly household income, No. (%)^b^	
<1200 €/mo	445 (15.8)
1200-2000 €/mo	377 (13.3)
>2000 €/mo	2003 (70.9)
Tobacco use during pregnancy, No. (%)	
Never	2160 (76.5)
Until pregnancy was known	253 (9.0)
Continued to smoke	412 (14.6)
Cannabis use, No. (%)	
Never	2669 (94.5)
Before pregnancy only	79 (2.8)
During pregnancy	77 (2.7)
Alcohol use during pregnancy, No. (%)	
Never	1044 (37.0)
Until pregnancy was known	396 (14.0)
Continued to drink, occasionally	1076 (38.1)
Continued to drink, frequently	309 (10.9)
Used antidepressant in pregnancy, No. (%)	24 (0.9)
Maternal depressive symptoms (Brief Symptom Inventory score), mean (SD)^c^	
20 wk of Gestation	0.20 (0.42)
2 mo Postnatally (n = 2004)	0.18 (0.44)
6 mo Postnatally (n = 1802)	0.22 (0.51)
Postnatal mean	0.20 (0.42)
**Child characteristics**	
Child sex, No. (%)	
Male	1329 (47.0)
Female	1496 (53.0)
Gestational age at birth, mean (SD), wk	39.9 (1.7)
Birth weight, mean (SD), g	3460.1 (557.5)
Child psychopathologic characteristics	
T1 (raw scores) (n = 1577)	
Child age at assessment, mean (SD), y	9.7 (0.01)
Internalizing symptoms, median (IQR)	2 (0-3)
Externalizing symptoms, median (IQR)	2 (0-3)
T2 (raw scores) (n = 1443)	
Child age at assessment, mean (SD), y	13.5 (0.02)
Internalizing symptoms, median (IQR)	7 (4-13)
Externalizing symptoms, median (IQR)	6 (3-9)
Child neuroimaging	
T1 (n = 1959)	
Child age, mean (SD), y	10.2 (0.6)
Framewise displacement, mean (SD) [range]	0.08 (0.03) [0.02-0.24]
Global efficiency, mean (SD)	0.36 (0.04)
Modularity, mean (SD)	0.22 (0.04)
Clustering coefficient, mean (SD)	0.16 (0.02)
T2 (n = 1668)	
Child age, mean (SD), y	13.9 (0.6)
Framewise displacement, mean (SD) [range]	0.07 (0.03) [0.02-0.23]
Global efficiency, mean (SD)	0.40 (0.05)
Modularity, mean (SD)	0.25 (0.5)
Clustering coefficient, mean (SD)	0.17 (0.3)

^a^ Pooled imputed data are shown (except for maternal depressive symptom and child psychopathology scores).

^b^ To convert euros to US dollars, multiply by 1.0875.

^c^ Scores range from 0 to 4, with higher scores indicating more severe depressive symptoms.

eTable 3 in Supplement 1 provides descriptive information on women with clinically relevant prenatal and postnatal depressive symptoms (prenatal, 221 [7.8%]; postnatal, 158 [5.6%]), as well as their respective reference groups. The exposure groups were comparable in key sociodemographic characteristics.

### Maternal Depressive Symptoms (Continuously Modeled)

#### Graph Theory Metrics

Table 2 shows associations between perinatal maternal depressive symptoms and repeated measures of graph metrics from midchildhood to adolescence. There was no overall association across time, meaning no association with brainwide functional architecture persisted across ages. Prenatal maternal depressive symptoms were associated with a greater increase in offspring brain global efficiency (interaction; β = 0.004, SE = 0.000; FDR-corrected *P* = .002) and modularity (interaction: β = 0.003, SE = 0.000; FDR-corrected *P* = .002). Prenatal maternal depressive symptoms were not associated with the clustering coefficient. Postnatal maternal depressive symptoms showed no association with any graph metrics.

**Table 2.** Association of Maternal Depressive Symptoms (Continuous) With Offspring Brainwide Functional Architecture

Effect	Symptoms^a^					
	Prenatal depression (n = 2825)			Postnatal depression (n = 2260)		
	Estimate (SE)	Unadjusted *P* value	FDR-corrected *P* value	Estimate (SE)	Unadjusted *P* value	FDR-corrected *P* value
**Global efficiency**						
Main effect	0.000 (0.002)	.67	.67	0.001 (0.002)	.48	.48
Interaction effect	0.004 (0.000)	<.001	.002^b^	0.003 (0.002)	.06	.12
**Modularity**						
Main effect	0.001 (0.002)	.56	.67	0.001 (0.002)	.40	.48
Interaction effect	0.003 (0.000)	<.001	.002^b^	0.003 (0.002)	.08	.12
**Clustering coefficient**						
Main effect	0.000 (0.001)	.64	.67	−0.004 (0.003)	.13	.39
Interaction effect	−0.003 (0.002)	.06	.12	0.000 (0.002)	.95	.95

Abbreviation: FDR, false discovery rate.

^a^ Linear mixed-effect models were used to test the associations of prenatal and postnatal exposure to maternal depressive symptoms (continuous) with repeatedly assessed brainwide functional architecture from ages 9 to 15 years. Effect estimates including the main effect and interaction effect (β) (metric change; interaction of depressive symptoms score × age), as well as SEs, unadjusted *P* values, and FDR-corrected *P* values, are shown. All models were adjusted for child sex, child age at the neuroimaging assessment, maternal age at intake, maternal national origin, marital status, maternal educationl level, maternal substance use (tobacco, cannabis, alcohol), monthly household income, and in-scanner head motion (mean framewise displacement). The postnatal depressive symptoms model was additionally adjusted for prenatal depression scores.

^b^ Indicates significant associations after FDR correction for multiple testing for graph theory metrics.

#### Within- and Between-Network Connectivity

eTable 7 in Supplement 1 shows the associations of prenatal and postnatal maternal depressive symptoms with the repeated measures of within- and between-network RSFC among offspring. Prenatal maternal depressive symptoms were associated with a greater increase in the RSFC of the within-network default mode network (DMN) with age (interaction: β = 0.010, SE = 0.001; FDR-corrected *P* = .002). Postnatal symptoms were not associated with any within-network RSFC. No associations were found between prenatal or postnatal symptoms and between-network RSFC after correction for multiple testing.

### Clinically Relevant Maternal Depressive Symptoms (Dichotomously Modeled)

**Figure 1.**
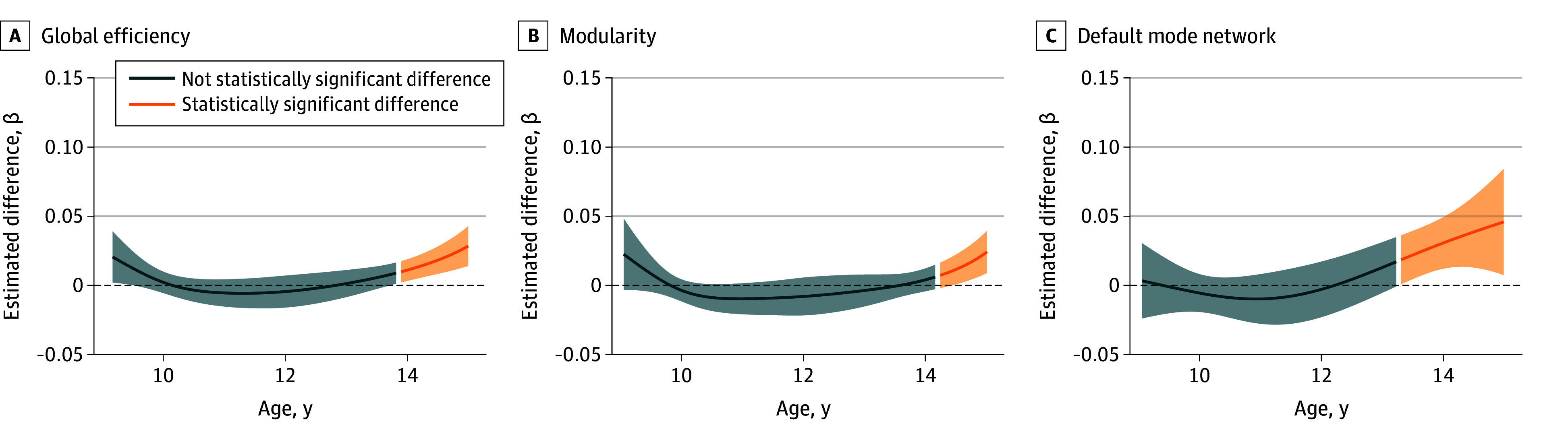
Estimated Relative Differences in Offspring’s Functional Network Connectivity Exposed to Clinically Relevant Prenatal Maternal Depressive Symptoms (n = 221) vs Reference (n = 2604) The associations of dichotomized maternal depressive symptoms are illustrated, although the text focuses on the main analyses using continuous depression scores. Estimated difference trajectories (as β) are shown with 95% CIs (shaded areas) for global efficiency, modularity, and within-network connectivity in the default mode network for clinically relevant prenatal maternal depressive symptoms at ages 9 to 15 years. All generalized additive mixed models were adjusted for child sex, child age at neuroimaging assessment, maternal age at intake, maternal national origin, marital status, maternal educational level, maternal substance use (tobacco, cannabis, alcohol), monthly household income, and in-scanner head motion (mean framewise displacement). Specific estimates from the generalized additive mixed models are shown in eTables 9 and 10 in Supplement 1.

To compare the functional brain connectivity trajectories of children exposed to clinically relevant prenatal and postnatal maternal depressive symptoms, we plotted the difference curves for each group compared with the reference group without clinically relevant symptoms (Figure 1). Model estimates and results from linear mixed-effects model and generalized additive mixed model models are provided in the eResults and eTables 6, 8, 9, and 10 in Supplement 1. No group differences emerged in childhood, but increased global efficiency, modularity (Figure 1A), and within-network DMN (Figure 1B) among prenatally exposed children became apparent from the age of 13 years onward.

**Figure 2.**
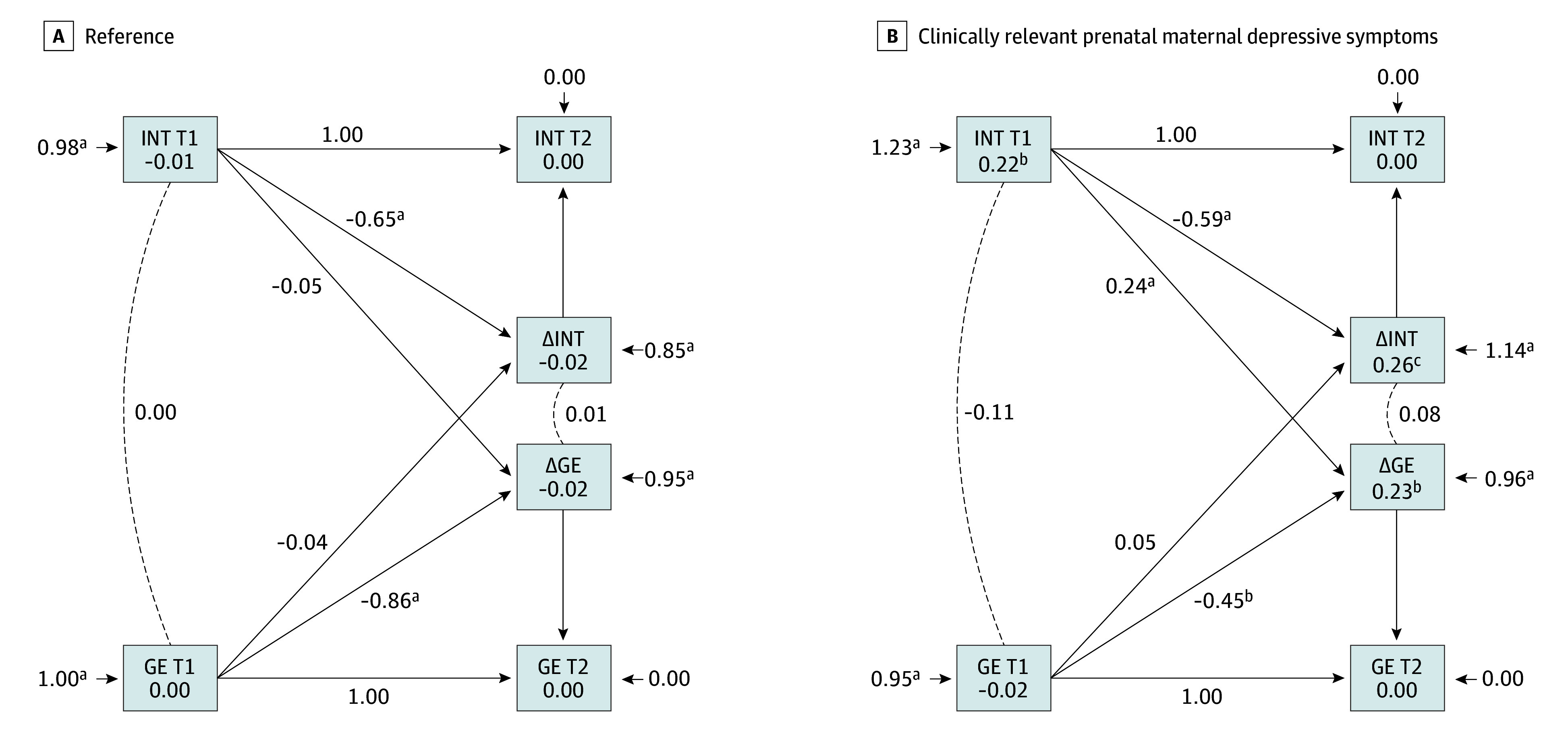
Graph Showing the Multigroup Bivariate Latent Change Score (BLCS) Model for Child-Reported Internalizing (INT) Problems and Global Efficiency (GE) Relations Standardized effects (as β) from the BLCS model are presented. T1 represents the baseline, and T2 is the follow-up score. ΔINT and ΔGE denote the change scores in INT and GE, respectively, between baseline and follow-up. The model was conducted within a multigroup framework, where the exposure status (reference [n = 2604] vs clinically relevant prenatal maternal depressive symptoms [n = 221]) served as the grouping variable. Models were adjusted for child sex, child age at the neuroimaging assessment, maternal age at intake, maternal national origin, marital status, maternal educational level, maternal substance use (tobacco, cannabis, alcohol), monthly household income, in-scanner head motion (mean framewise displacement), and age difference between the behavioral and resting-state functional magnetic resonance imaging assessment. The dashed lines represent baseline associations between the 2 constructs (eg, INT T1 and GE T1), as well as the co-occurrence of changes in these measures over time. In contrast, the solid lines indicate directional predictive paths from baseline measures to change scores and subsequent time points within the BLCS model framework. ^a^*P* < .001. ^b^*P* < .05. ^c^*P* < .01.

Internalizing problems and brain metrics results are shown in Figure 2 and eTable 11 in Supplement 1. No associations were found at baseline or in overall change rates. However, more severe baseline internalizing problems were associated with steeper increases in global efficiency over time among those exposed to clinically relevant prenatal maternal depressive symptoms (β = 0.243, SE = 0.37; FDR-corrected *P* = .001). The χ^2^ tests revealed a significant group difference (χ^2^_1_ = 7.2; *P* = .007); the association between baseline internalizing problems and the change in global efficiency was stronger among children exposed to clinically relevant prenatal maternal depressive symptoms than those not exposed. eFigures 5 and 6 in Supplement 1 illustrate the associations between internalizing problems, modularity, and within-network DMN. No associations were found between externalizing problems and any brain metric (eFigures 7, 8, and 9 in Supplement 1).

## Discussion

In this population-based cohort study from fetal life onward, more severe prenatal maternal depressive symptoms were associated with steeper increases in global efficiency, modularity, and within-network DMN connectivity, associations emerging only in adolescence. Postnatal symptoms showed no associations. Internalizing problems at baseline were associated with greater increases in global efficiency among those exposed to clinically relevant prenatal maternal depressive symptoms.

To our knowledge, no study has examined the association between exposure to maternal depressive symptoms and change in functional brain connectivity among offspring. During normative development, global efficiency of brain networks increases from childhood to adolescence, which reflects the brain’s ability to integrate information across distributed regions.^38^ This transition period is characterized by heightened segregation alongside enhanced local clustering and modularity, indicating a shift in brain network organization from a locally focused architecture among children to a more distributed pattern among young adults.^39^ Previous studies have highlighted alterations in global brain networks across mood and neurodevelopmental disorders, underscoring the importance of understanding the differences in brain network connectivity among these conditions.^40,41^ Although speculative, the concurrent increases in global efficiency and modularity observed in our study suggest that prenatal maternal depressive symptoms may be selectively associated with the development of large-scale brain network architecture. This dual enhancement is likely associated with the DMN’s long-distance connections, which uniquely support both integration and segregation.^42^ Although these changes may reflect adaptive brain development, they could also predispose offspring to mental disorders later in life.^6,43^

Our findings broadly align with the stress acceleration hypothesis, which posits that early adversity may expedite biological maturation as a short-term adaptive response.^44^ However, the observed associations, emerging predominantly in midadolescence, potentially suggest a more nuanced developmental alteration, reflecting an exaggerated or delayed reorganization of functional networks. This pattern is consistent with models of stage-specific recalibration, in which early adversity alters the trajectory rather than the absolute timing of brain development.^45^ Functional network segregation and integration follow a local-to-global developmental sequence, with regional clustering maturing earlier and global properties, such as modularity and efficiency, emerging later.^46^ The observed associations may reflect this hierarchical progression, with group-level differences becoming detectable only once higher-order global organization is sufficiently established. Neurobiological processes, such as altered synaptic pruning, myelination, or excitatory–inhibitory balance, may underlie this pattern, whereby early exposures shape the architecture of functional brain networks that mature more during adolescence.^45^

Furthermore, child internalizing problems may precede or reflect functional network integration among individuals exposed to clinically relevant prenatal maternal depressive symptoms. Specifically, internalizing problems were associated with steeper increases in global efficiency among exposed youths, suggesting a potential coupling between emotional functioning and large-scale network organization. Although such changes may reflect accelerated maturation, they could also indicate reduced neurodevelopmental plasticity, particularly if they deviate from the more protracted, locally driven trajectory typically associated with enriched learning and adaptability during adolescence.^38,44,45,47^ Premature shifts in global integration may therefore limit the brain’s capacity for flexible reorganization during this sensitive developmental window.^38,44,45,47^ The fact that internalizing problems temporally preceded observable changes in network architecture highlights the importance of further research into whether early behavioral interventions could help normalize the pace of functional brain maturation and mitigate long-term mental health risks.

In addition to the graph theory measures, analyzing within- and between-network RSFC allowed us to investigate the associations between more fine-grained connectivity networks. We found prenatal maternal depressive symptoms were associated with a steeper increase in within-network DMN, a network associated with attention to internal states and self-referential thinking, among offspring specifically.^48^ Previous studies have shown that the DMN follows a distinct developmental trajectory, with initially weak connections that strengthen significantly by adolescence and may be particularly sensitive to early-life adversities.^15,49^ Moreover, increased connectivity within the DMN has been associated with early-onset depression, highlighting its clinical significance.^50^ The selective heightened within-network DMN connectivity observed in this study may also explain the concurrent increases in global efficiency and modularity, as the DMN’s long-distance connections between distributed brain regions are associated with enhanced integration and segregation at the network level.^15,46^ Within this context, the alterations in within-network DMN connectivity associated with prenatal depression exposure could reflect an increased risk for depression in adolescence. As age-related increases in internalizing and other mental health problems continue to emerge,^51^ it will be important to explore whether heightened within-network DMN connectivity is prospectively associated with the onset of psychopathologic conditions.

The lack of associations between postnatal maternal depressive symptoms and differences in functional brain connectivity cannot easily be compared with prior research. The pattern of an association of prenatal rather than postnatal maternal depressive symptoms with functional connectivity in the cortico-amygdala-striatal circuitry has been reported previously, but in a study of girls in early childhood.^52^ Here, associations of postnatal maternal depressive symptoms with medial orbitofrontal connectivity had disappeared by 6 years of age.^53^ However, the major methodological differences in age, study design, and assessments allow for cautious comparisons only. Our findings may reflect a heightened developmental sensitivity to intrauterine exposures.

Several mechanisms may underlie these associations. Clinically relevant maternal symptoms may co-occur with pregnancy complications, nutritional deficits, or stress-reactive physiology, all of which can affect neurodevelopment.^9^ In addition, prenatal depression may alter DNA methylation in key stress-related genes (eg, *NR3C1* [OMIM 600535], *BDNF* [OMIM 113505]), with lasting associations with brain development.^9^ Genetic predispositions to depression may be associated with differences in offspring’s brain connectivity.^43,54^

### Limitations

This study has some limitations. First, while the Brief Symptom Inventory serves as a validated tool for assessing psychopathologic conditions in large-scale studies, it is less commonly used for perinatal maternal depression. Second, the relatively low prevalence of depression within our study population could have constrained our ability to detect associations, particularly in the dichotomized analyses, despite the large sample size. Consequently, the results must be interpreted cautiously, especially when generalizing them to more vulnerable populations.

## Conclusions

In this cohort study, prenatal maternal depressive symptoms were associated with altered trajectories of functional connectivity during adolescence. Internalizing problems were associated with greater increases in global efficiency, suggesting that these problems may be associated with brain maturation processes. These findings highlight prenatal maternal depression as a sensitive window that may be associated with emotional problems and neurodevelopment, with potential implications for prevention and early intervention.

## Notes

Supplement 1.
eMethods.

eResults.
**eFigure 1.** Schematic Overview of the Study **eFigure 2.** Flow Diagram of the Study Population **eFigure 3.** Showing the Age of Each Participant at Each Study Time-Point **eFigure 4.** Within- and Between-Network Resting-State Functional Connectivity (RSFC) Across Time Points **eFigure 5.** Graph Showing the Multigroup Bivariate Latent Change Score (BLCS) Model for Child-Reported Internalizing (INT) Problems and Modularity (MOD) Relations **eFigure 6.** Graph Showing the Multigroup Bivariate Latent Change Score (BLCS) Model for Child-Reported Internalizing (INT) Problems and Default Mode Network (DMN) Relations **eFigure 7.** Graph Showing the Multigroup Bivariate Latent Change Score (BLCS) Model for Child-Reported Externalizing (EXT) Problems and Global Efficiency (GE) Relations **eFigure 8.** Graph Showing the Multigroup Bivariate Latent Change Score (BLCS) Model for Child-Reported Externalizing (EXT) Problems and Modularity (MOD) Relations **eFigure 9.** Graph Showing the Multigroup Bivariate Latent Change Score (BLCS) Model for Child-Reported Externalizing (EXT) Problems and Default Mode Network (DMN) Relations **eTable 1.** Number of Images of the Participants in Each Group (Prenatal Depression vs Reference) at T1 and T2 **eTable 2.** Number of Images of the Participants in Each Group (Postnatal Depression vs Reference) at T1 and T2 **eTable 3.** Demographic Characteristics of the Study Population for Exposure Groups **eTable 4.** Non-Response Analysis **eTable 5.** Comparison of Results From the Unweighted Model With Inverse Probability of Attrition Weighting (IPAW) Models **eTable 6.** Association of Clinically Relevant Maternal Depressive Symptoms With Offspring Functional Network Connectivity (Graph Theory Measures) **eTable 7.** Association of Maternal Depressive Symptoms (Continuous) With Offspring Within- and Between-Network Functional Connectivity **eTable 8.** Association of Clinically Relevant Maternal Depressive Symptoms With Offspring Within- and Between-Network Functional Connectivity Using the Gordon Parcellation **eTable 9.** Association of Clinically Relevant Maternal Depressive Symptoms With Offspring Functional Network Connectivity (Graph Theory Measures) **eTable 10.** Association of Clinically Relevant Maternal Depressive Symptoms With Offspring Within- and Between-Network Functional Connectivity Using the Gordon Parcellation **eTable 11.** Multigroup BLCS Model for Each Brain rsfMRI Metric
eReferences.

Supplement 2.
Data Sharing Statement
