# Supplementary material for: Perinatal Maternal Depressive Symptoms and Brain Connectivity Among 9- to 15-Year-Old Offspring
Source: JAMA Netw Open. 2025 Jul 31;8(7):e2523978. doi: 10.1001/jamanetworkopen.2025.23978 (PMC12314728; doi:10.1001/jamanetworkopen.2025.23978)
Supplement: Supplement 1. — eMethods. eResults. eFigure 1. Schematic Overview of the Study eFigure 2. Flow Diagram of the Study Population eFigure 3. Showing the Age of Each Participant at Each Study Time-Point eFigure 4. Within- and Between-Network Resting-State Functional Connectivity (RSFC) Across Time Points eFigure 5. Graph Showing the Multigroup Bivariate Latent Change Score (BLCS) Model for Child-Reported Internalizing (INT) Problems and Modularity (MOD) Relations eFigure 6. Graph Showing the Multigroup Bivariate Latent Change Score (BLCS) Model for Child-Reported Internalizing (INT) Problems and Default Mode Network (DMN) Relations eFigure 7. Graph Showing the Multigroup Bivariate Latent Change Score (BLCS) Model for Child-Reported Externalizing (EXT) Problems and Global Efficiency (GE) Relations eFigure 8. Graph Showing the Multigroup Bivariate Latent Change Score (BLCS) Model for Child-Reported Externalizing (EXT) Problems and Modularity (MOD) Relations eFigure 9. Graph Showing the Multigroup Bivariate Latent Change Score (BLCS) Model for Child-Reported Externalizing (EXT) Problems and Default Mode Network (DMN) Relations eTable 1. Number of Images of the Participants in Each Group (Prenatal Depression vs Reference) at T1 and T2 eTable 2. Number of Images of the Participants in Each Group (Postnatal Depression vs Reference) at T1 and T2 eTable 3. Demographic Characteristics of the Study Population for Exposure Groups eTable 4. Non-Response Analysis eTable 5. Comparison of Results From the Unweighted Model With Inverse Probability of Attrition Weighting (IPAW) Models eTable 6. Association of Clinically Relevant Maternal Depressive Symptoms With Offspring Functional Network Connectivity (Graph Theory Measures) eTable 7. Association of Maternal Depressive Symptoms (Continuous) With Offspring Within- and Between-Network Functional Connectivity eTable 8. Association of Clinically Relevant Maternal Depressive Symptoms With Offspring Within- and Between-Network Functional Connectivity Using [file jamanetwopen-e2523978-s001.pdf]

Supplementary Online Content

Koc D, Hermans APC, Xu B, Muetzel RL, El Marroun, Tiemeier H. Perinatal maternal depressive symptoms and brain connectivity among 9- to 15-year-old offspring. *JAMA Netw Open.* 2025;8(7):e2523978. doi:10.1001/jamanetworkopen.2025.23978

eMethods.

eResults.

eFigure 1. Schematic Overview of the Study

eFigure 2. Flow Diagram of the Study Population

eFigure 3. Showing the Age of Each Participant at Each Study Time-Point

eFigure 4. Within- and Between-Network Resting-State Functional Connectivity (RSFC) Across Time Points

eFigure 5. Graph Showing the Multigroup Bivariate Latent Change Score (BLCS) Model for Child-Reported Internalizing (INT) Problems and Modularity (MOD) Relations

eFigure 6. Graph Showing the Multigroup Bivariate Latent Change Score (BLCS) Model for Child-Reported Internalizing (INT) Problems and Default Mode Network (DMN) Relations

eFigure 7. Graph Showing the Multigroup Bivariate Latent Change Score (BLCS) Model for Child-Reported Externalizing (EXT) Problems and Global Efficiency (GE) Relations

eFigure 8. Graph Showing the Multigroup Bivariate Latent Change Score (BLCS) Model for Child-Reported Externalizing (EXT) Problems and Modularity (MOD) Relations

eFigure 9. Graph Showing the Multigroup Bivariate Latent Change Score (BLCS) Model for Child-Reported Externalizing (EXT) Problems and Default Mode Network (DMN) Relations

eTable 1. Number of Images of the Participants in Each Group (Prenatal Depression vs Reference) at T1 and T2

eTable 2. Number of Images of the Participants in Each Group (Postnatal Depression vs Reference) at T1 and T2

eTable 3. Demographic Characteristics of the Study Population for Exposure Groups

eTable 4. Non-Response Analysis

eTable 5. Comparison of Results From the Unweighted Model With Inverse Probability of Attrition Weighting (IPAW) Models

eTable 6. Association of Clinically Relevant Maternal Depressive Symptoms With Offspring Functional Network Connectivity (Graph Theory Measures)

© 2025 Koc D et al. *JAMA Network Open.*

eTable 7. Association of Maternal Depressive Symptoms (Continuous) With Offspring Within- and Between-Network Functional Connectivity

eTable 8. Association of Clinically Relevant Maternal Depressive Symptoms With Offspring Within- and Between-Network Functional Connectivity Using the Gordon Parcellation

eTable 9. Association of Clinically Relevant Maternal Depressive Symptoms With Offspring Functional Network Connectivity (Graph Theory Measures)

eTable 10. Association of Clinically Relevant Maternal Depressive Symptoms With Offspring Within- and Between-Network Functional Connectivity Using the Gordon Parcellation

eTable 11. Multigroup BLCS Model for Each Brain rsfMRI Metric

eReferences.

This supplementary material has been provided by the authors to give readers additional information about their work.

© 2025 Koc D et al. *JAMA Network Open.*

eMethods.

Clinically relevant maternal depressive symptoms

Brief Symptom Inventory (BSI)[1] is a validated self-report questionnaire for maternal psychopathology, showing good internal consistency in our sample (Cronbach's alpha ranged from 0.82 to 0.88 across assessments). For mothers who reported depressive symptoms at both 2 and 6 months postpartum, scores were averaged.

In a subgroup of 905 women from the Generation R cohort, we evaluated the performance of the BSI in detecting clinical depression using the recommended cut-off score.[2] To assess clinical depression in this subgroup, we used the Composite International Diagnostic Interview (CIDI), which is a structured interview based on DSM-IV criteria and has been reported to have good reliability and validity.[3] We calculated the positive likelihood ratio (LR+) as it is more suitable for detecting low prevalence conditions, and the calculated LR+ was 7.29.[2] This indicates that the cut-off score has moderate diagnostic accuracy in identifying clinical depression. Additionally, in the same subgroup, we assessed the BSI's ability to detect postpartum depression. Data on postpartum depression was collected using the Edinburgh Postnatal Depression Scale (EPDS), a validated 10-item self-report scale commonly used in the Netherlands.[4] EPDS sum scores range from 0 to 30, with higher scores indicating more depressive symptoms. We used the validated EPDS cut-off score of over 12. In a community sample, this cut-off score has a sensitivity of over 80% and a specificity of 95% for identifying women with clinically diagnosed postpartum depression.[4] We calculated LR+ as 28.68. This demonstrates the good quality of the BSI cut-off as an indicator of depression as measured with the EPDS.[2]

© 2025 Koc D et al. *JAMA Network Open.*

Based on the depressive symptoms, pregnant mothers were classified;

1. Prenatal depression classification;

(I) **Reference (n = 2604):** Women who had a score lower than clinically significant depressive symptoms during pregnancy (20 weeks gestation);

(II) **Clinically relevant prenatal depressive symptoms (n = 221):** women who had clinically significant depressive symptoms during pregnancy (20 weeks gestation);

2. Postnatal depression classification;

(I) **Reference (n = 2102):** Women who had a score lower than clinically significant depressive symptoms in the postnatal period (2 and/or 6 months postnatally);

(II) **Clinically relevant postnatal depressive symptoms (n = 158** women who had clinically relevant depressive symptoms in the postnatal period (2 and/or 6 months postnatally).

Prenatal antidepressant use

Information was obtained from self-reported questionnaires and pharmacy prescriptions to optimally assess antidepressant use during pregnancy. Mothers were asked if they had used medications in the preceding 6 months throughout the first trimester. In the second and third trimester, we asked which medications were used in the previous three months. We assessed any antidepressant intake and timing using these questionnaires (before or during pregnancy). To validate self-report of antidepressant prescriptions, we used pharmacy data to verify antidepressant use which showed high agreement with self-reported use: Yule's Y was 0.94.[2,5] More details regarding maternal antidepressant use can be found elsewhere.[2,6,7]

© 2025 Koc D et al. *JAMA Network Open.*

Child Problem Behavior

At T1, children completed the validated Brief Problem Monitor (BPM), and at T2, they filled out the Youth Self Report (YSR). Both assessments were rated on a 3-point scale (0 = not true, 1 = somewhat or sometimes true, 2 = very or often true). Both the BPM and YSR have demonstrated good reliability and validity.[8]

Image acquisition and pre-processing

Resting-state fMRI scans were obtained with an interleaved axial echo planar imaging sequence with 200 volumes. A repetition time of 1,760 milliseconds (ms), echo time of 30 ms, flip angle of 85 degrees, a 64x64 matrix, field of view of 230x230 millimeters (mm), and slice thickness of 4 mm were used.[9] Further details on the scanning procedure can be found elsewhere.[9]

Motion-related artifacts were addressed using ICA-AROMA,[10] nuisance signals from white matter and cerebrospinal fluid (CSF) were regressed out, and the data underwent high-pass filtering to remove signals below 0.01 Hz. Global signal regression was not applied in our analyses due to ongoing debate over its utility and potential drawbacks. While global signal regression can effectively reduce noise, it also risks removing meaningful neural fluctuations alongside non-neural sources, such as respiratory and cardiac activity.[11]

Quality control and motion correction

Scans with major artifacts, inaccurate co-registration to MNI, insufficient whole-brain coverage (e.g., missing large portions of the cerebrum or cerebellum from the field of view), and excessive motion (more than 20% of the volumes with a framewise displacement higher than 0.2 mm or a mean framewise displacement higher than 0.25 mm) were excluded. Moreover, the accuracy of

© 2025 Koc D et al. *JAMA Network Open.*

co-registration was visually inspected by merging all co-registered images into a single 4D Nifti image and scrolling through the images.[12]

Additionally, ICA-AROMA was applied at the subject level to remove motion-related artifacts before constructing subject-level connectivity matrices and networks.[10] To further account for residual motion effects, in-scanner head motion (mean framewise displacement) was included as a technical covariate in all statistical models.

Covariates

Maternal national origin was defined according to the classification of Statistics Netherlands: Dutch, non-Dutch European (including European and North American countries). The non-European group was further divided into four subgroups: Caribbean (Dutch Antillean, Surinamese, and South American), Moroccan/Turkish (Moroccan and Turkish), African (Cape Verdean and other African), and Asian Oceanian (Indonesian) mothers. Maternal age at prenatal intake was self-reported, and maternal education was classified as low (no education/primary school only), secondary (high school/vocational training), and high (higher vocational training/university). Parents reported the household income during pregnancy as less than €1200,- (below poverty level), €1200-€2000,- (low income in 2005), or more than €2000,- per month. Questionnaires were used to assess maternal tobacco use, categorized into three categories: never, until pregnancy was known, and continued during pregnancy. For cannabis use data was collected in the first trimester and validated with urine samples: never, before pregnancy only, and during pregnancy. Alcohol use during pregnancy was categorized as never, until pregnancy was known, continued to drink occasionally during pregnancy, and continued to drink frequently (one or more glasses of alcohol per week in at least two trimesters).

© 2025 Koc D et al. *JAMA Network Open.*

Statistical analysis

Linear Mixed-Effects Models (LMMs)

Given the unbalanced design, where the number of participants differed between exposure and reference groups, models were estimated using restricted maximum likelihood (REML). P-values were derived using the Kenward–Roger approximation via the lmerTest package, which provides appropriate degrees of freedom correction and Type I error control in unbalanced, longitudinal designs.[6,7]

Generalized additive mixed model (GAMM)

To model possible non-linear trajectories of functional brain development, a smoothing function was applied to the age of the child (predictor) in a GAMM. We opted for smooth splines to capture better essential nonlinear changes that traditional polynomials might miss. We selected the number of basis smooth functions (k parameters) based on results from k.check (mgcv package) analyses of the models to counteract a potential overfit,[18] resulting in four smooth functions. To account for longitudinal data, we also included a smoothing function, equivalent to adding a random effect in LME, for each subject. GAMM was run with individual trajectories estimated for each exposure group and reference classification (**eFigure 1C.1**) using this formula:

Outcome ~ group + s(age, by = group) + covariates + s(subject, bs = "re")

where group indicates clinically relevant depressive symptom status (binary), s(age, by = group) specifies separate smooth terms for each group, and s(subject, bs = "re") includes random intercepts for individuals to account for repeated measures.

© 2025 Koc D et al. *JAMA Network Open.*

RSFC trajectories and estimated difference curves were plotted only if a significant interaction between the exposure group and age was observed. These curves were generated using the 'gratia' package to assess exposure group trajectories (e.g., clinically relevant maternal depressive symptoms vs. reference). Trajectories where the confidence interval did not include zero were considered areas of significant change.

Bivariate Latent Change Score (BLCS) Model

The BLCS model was used to investigate the dynamic interplay between maternal depressive symptoms, child internalizing problems, and brain functional connectivity networks over time. This approach is particularly suited for exploring longitudinal relationships as it simultaneously evaluates changes in two interrelated variables and examines how these changes influence each other at successive time points.[19]

The BLCS model captures latent change scores, representing unobserved changes in each variable over time (**Figure 1C.2**). By incorporating coupling parameters, the model quantifies the extent to which changes in one variable are influenced by the prior levels of the other variable, providing insights into directional relationships. Additionally, autoregressive parameters estimate the stability of each variable over time by assessing how its prior levels predict subsequent changes.

In this study, the BLCS model was applied within a multigroup framework to account for group differences based on exposure to clinically relevant maternal depressive symptoms. The primary objectives of the analysis were to examine baseline associations between child internalizing problems and brain connectivity metrics, determine whether baseline levels of brain metrics predicted changes in internalizing problems (and vice versa), and evaluate whether changes in

internalizing problems and brain connectivity metrics co-occurred over time. By assessing these relationships within a unified framework, the BLCS model enabled us to identify dynamic interactions between behavioral symptoms and functional connectivity.

To evaluate whether maternal depressive symptoms altered the temporal dynamics, Bentler-scaled χ^2-difference tests[20] were employed to assess group differences, with externalizing problems included as a contrasting exposure to contextualize the findings. The multigroup framework facilitated the comparison of pathways across exposure groups. Full information maximum likelihood estimation procedures were employed in the BLCS analysis.

Non-response and sensitivity analyses

We used chi-square tests for categorical variables and t-tests or Wilcoxon tests for continuous variables in non-response analyses. Given the differences in multiple characteristics between study population participants and those lost to follow-up (**eTable 4**), we used inverse probability of attrition weighting (IPAW) to account for potential selection bias in LME models.[21] In the sensitivity analysis, we excluded offspring exposed to antidepressants in utero (n = 24) as prenatal antidepressant exposure might alter offspring brain development differently than maternal depression.[22] As sex differences in prenatal programming are frequently observed, we conducted additional analyses to explore interactions based on the child's sex.[23]

Imputation and multiple testing correction

Multiple imputation by chained equations (using the ‘mice’ package)[24] was employed to address missing covariate data, with the highest proportion of missing values observed for family household income (18.7%) (eTable 4). 25 iterations and 25 imputed datasets were generated, and the results were pooled using Rubin’s rules.[25]

© 2025 Koc D et al. *JAMA Network Open.*

To minimize type I errors, false discovery rate (FDR) correction was applied using the Benjamini-Hochberg method.[26] This was performed separately for each hierarchical set of analyses: graph theory measures (3 comparisons) for the primary analyses, functional connectivity networks (24 comparisons, including within- and between-network connectivity) for the secondary analyses, and multigroup BLCS analyses (12 comparisons for the internalizing scale).

© 2025 Koc D et al. *JAMA Network Open.*

eResults.

Clinically relevant maternal depressive symptoms (dichotomously modeled)

Figure 1A depicts the estimated differences in trajectories of global graph metrics from ages 9-15 years. Compared to the reference, exposure to clinically relevant prenatal depressive symptoms was associated with a greater increase in functional connectivity of global efficiency (LME: group-by-age interaction, β = 0.004, *SE* = 0.001, P_{FDR} = .01; GAMM: group-by-s(age) interaction**,** *F* = 14.5, P_{FDR} = <.001, age range; 13.8-15) and modularity (LME: group-by-age interaction, β = 0.010, *SE* = 0.003, P_{FDR} = .006; GAMM: group-by-s(age) interaction**,** *F* = 8.1, P_{FDR} = <.001, age range; 14.2-15) (**Figure 1A, eTable 6 and eTable 9**). No associations of postnatal exposure to clinically relevant depressive symptoms with global graph metrics were observed (**eTable 6, eTable 9**).

Figure 1B depicts the estimated differences in trajectories of within-network DMN from ages 9-15 years. Compared to the reference, exposure to clinically relevant prenatal depressive symptoms was associated with a greater increase in RSFC of within-network DMN (LME: group-by-age interaction, β = 0.011, *SE* = 0.003, P_{FDR} = .004; GAMM: group-by-s(age) interaction**,** *F* = 18.6, P_{FDR} = <.001, age range; 13.2-15) (**Figure 1B, eTable 8 and eTable 10**). No associations between postnatal exposure to clinically relevant depressive symptoms and within and between-network RSFC were observed (**eTable 8, eTable 10**).

Non-response and sensitivity analyses

eTable 4 shows that compared to responders (n=3814), non-responding women (n=2825) were younger, more likely to be of non-Dutch origin, less educated, and more often smoked tobacco during pregnancy. The results of IPAW-weighted models did not differ from the non-weighted

© 2025 Koc D et al. *JAMA Network Open.*

models (**eTable 5**). Results did not change when participants exposed to antidepressants (n = 24) were excluded from the analysis and there was no evidence of interaction by child sex in the associations (data not shown).

eFigure 1. Schematic Overview of the Study

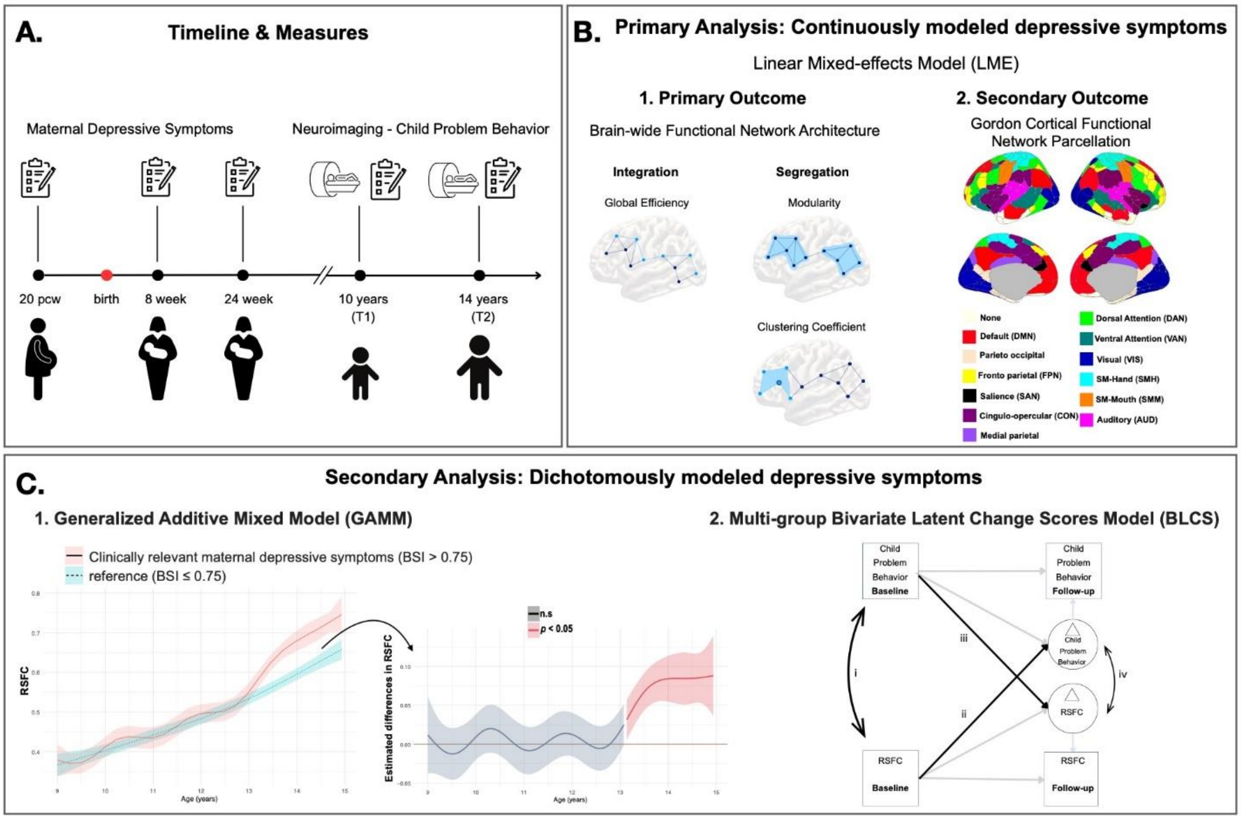

Note: **(A)** Timeline of assessments, including maternal depressive symptoms (prenatal and postnatal), child problem behavior, and neuroimaging sessions (T1 and T2, at mean ages 10 and 14 years, respectively). **(B)** Primary analyses used continuous measures of maternal depressive symptoms in linear mixed-effects models (LME) to assess associations with brain-wide functional architecture and specific functional networks across time. Primary outcomes included graph theory metrics—network integration (global efficiency) and network segregation (modularity, clustering coefficient)—offering insights into the organization and brain-wide functional network architecture (**1**). Secondary outcomes include resting-state functional connectivity (RSFC) networks based on the Gordon parcellation. AUD, auditory network; CON, cingulo-opercular network; DAN, dorsal attention network; DMN, default mode network; FPN, frontoparietal network; PON, parieto-occipital network; MPN, medial parietal network; SAN, salience network; SMH, sensorimotor hand network; SMM, sensorimotor mouth network; VAN, ventral attention network; VIS, visual network (**2**). **(C)** Secondary analyses involved dichotomizing maternal depressive symptoms into clinically relevant symptoms versus a non-exposed reference group. Generalized Additive Mixed Models (GAMM) were used to examine the developmental trajectories of RSFC across these groups. This panel illustrates the analytic concept using simulated data (**1**). The multigroup Bivariate Latent Change Scores (BLCS) model was applied to explore the dynamic relationship between child problem behavior and RSFC (**2**). Specifically, the BLCS model assessed (i) the relationship between child problem behavior and RSFC at baseline, (ii) whether RSFC at baseline predicts changes (Δ) in child problem behavior, (iii) whether baseline problem behavior predicts changes in RSFC, and (iv) the co-occurrence of changes in child problem behavior and RSFC over time.

© 2025 Koc D et al. *JAMA Network Open.*

eFigure 2. Flow Diagram of the Study Population

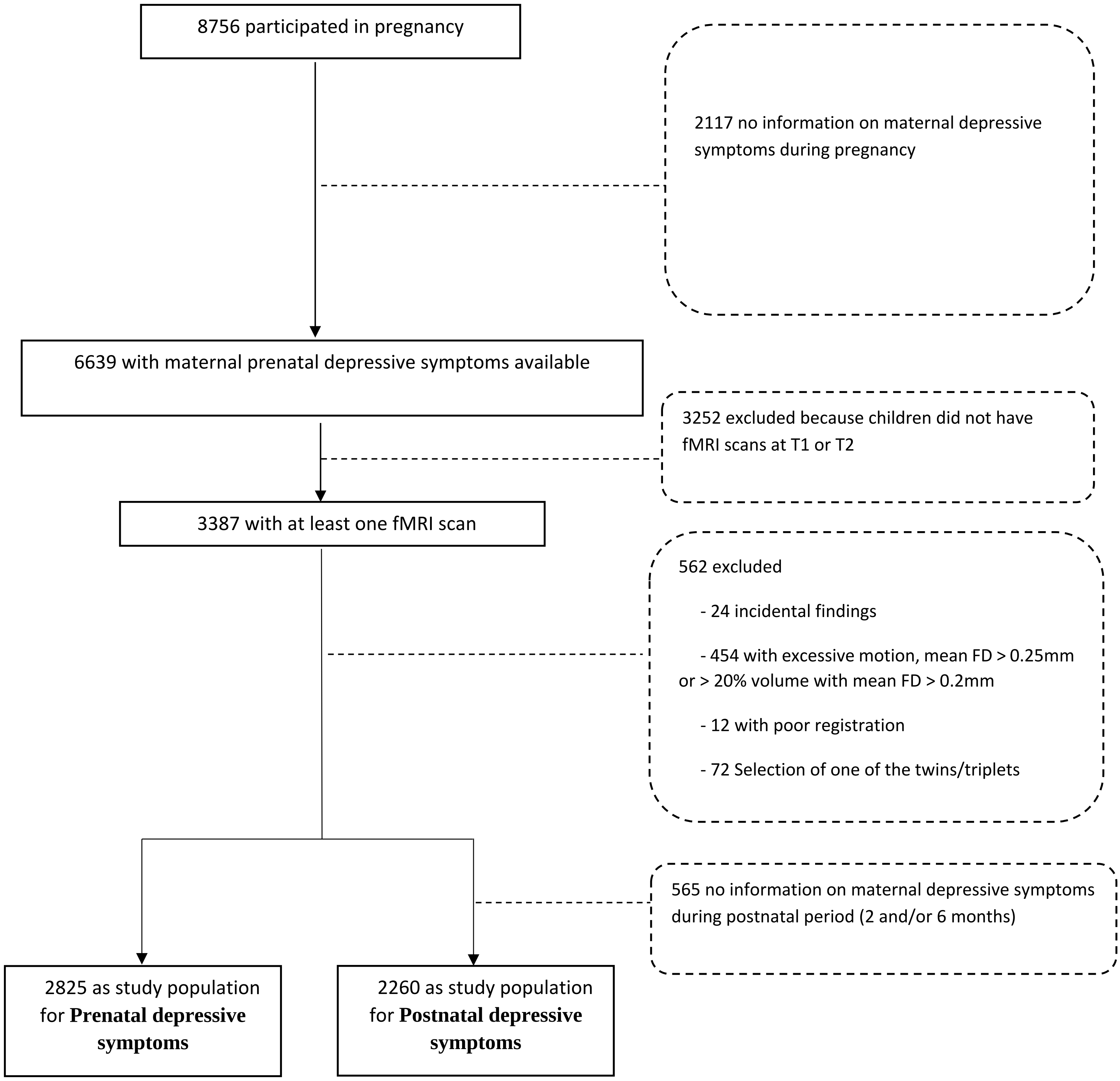

© 2025 Koc D et al. *JAMA Network Open.*

eFigure 3. Showing the Age of Each Participant at Each Study Time-Point

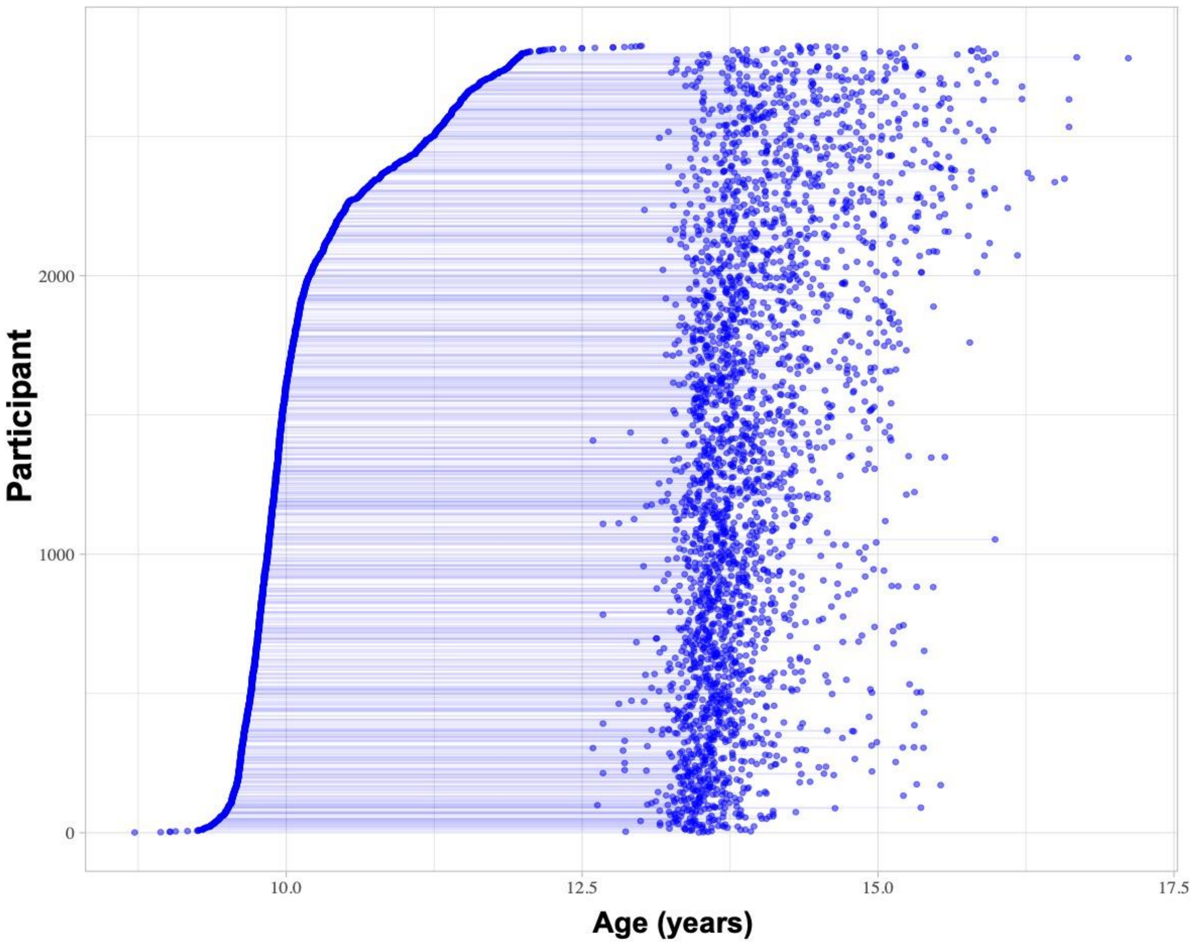

Note: A circle represents the 3627 acquired scans at T1 and T2, while each of the 2825 subjects is depicted as a separate row. A straight line connects the scans of individuals who underwent brain imaging at both time points.

eFigure 4. Within- and Between-Network Resting-State Functional Connectivity (RSFC) Across Time Points

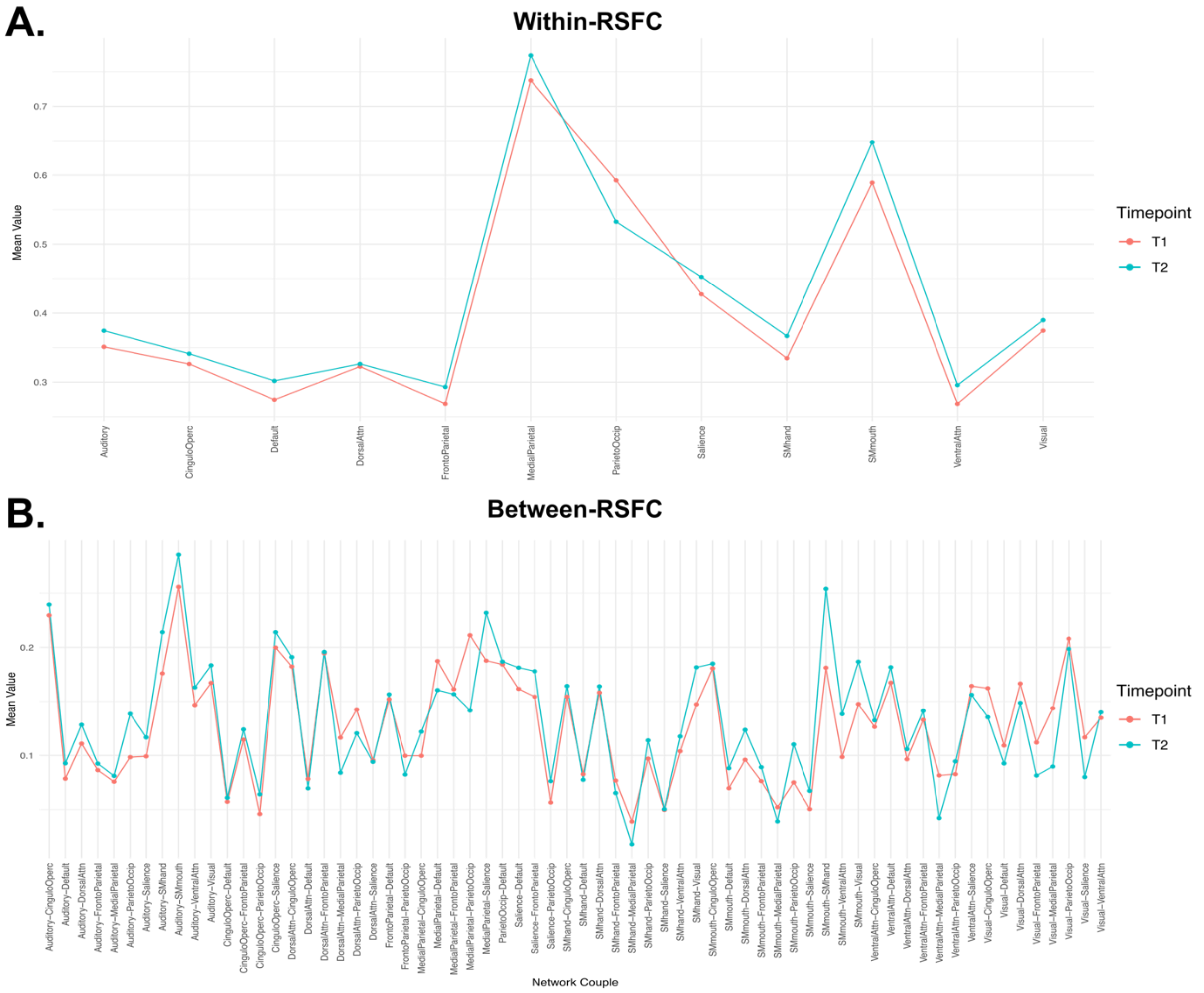

Note: Data are presented as mean values for each network or network pair, with T1 (red) and T2 (blue) representing the two-time points. Panel A shows the mean within-network RSFC values for 12 major brain networks (e.g., Default Mode, Sensorimotor, and Salience) at two-time points (T1 and T2). Panel B displays the mean between-network RSFC values for network pairs, with comparisons between the two-time points.

eFigure 5. Graph Showing the Multigroup Bivariate Latent Change Score (BLCS) Model for Child-Reported Internalizing (INT) Problems and Modularity (MOD) Relations

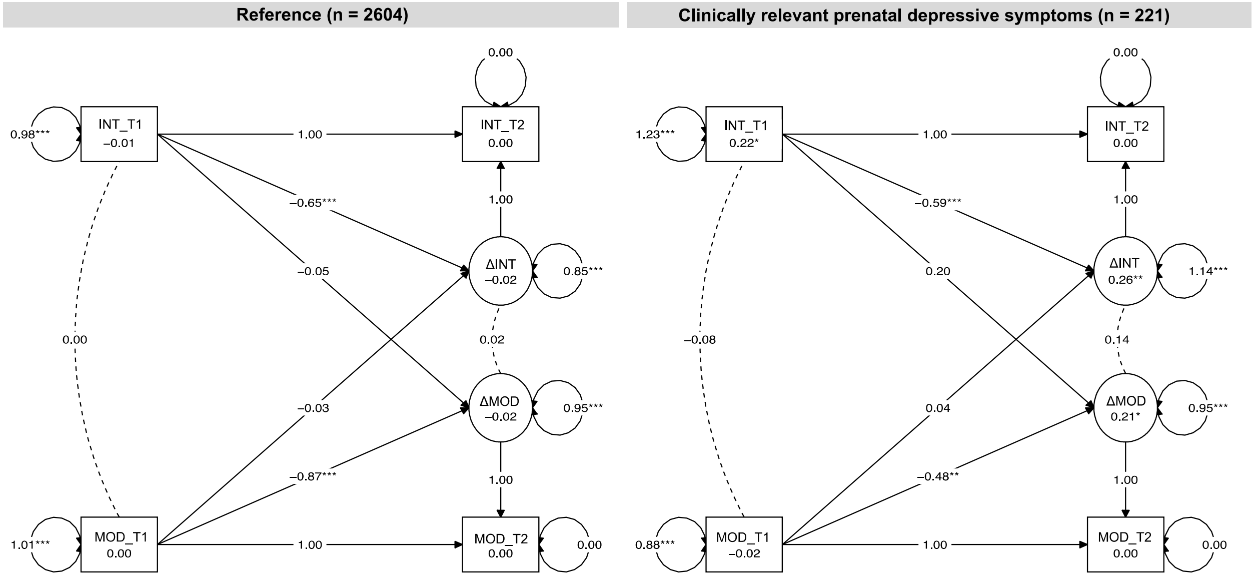

Note: Standardized effects from the BLCS model were presented. T1 represents the baseline, and T2 is the follow-up score. dINT and dMOD denote the change scores in INT and MOD between baseline and follow-up, respectively. The model was conducted within a multi-group framework, where the exposure status (reference vs. clinically relevant prenatal depressive symptoms) served as the grouping variable. Models were adjusted for child sex, child age at the neuroimaging assessment, maternal age at intake, maternal national origin, marital status, maternal education level, maternal substance use (tobacco, cannabis, alcohol), monthly household income, in-scanner head motion (mean framewise displacement), and the age difference between the behavioral and rsfMRI assessment.

* p < .05; ** p < .01; *** p < .001

© 2025 Koc D et al. *JAMA Network Open.*

eFigure 6. Graph Showing the Multigroup Bivariate Latent Change Score (BLCS) Model for Child-Reported Internalizing (INT) Problems and Default Mode Network (DMN) Relations

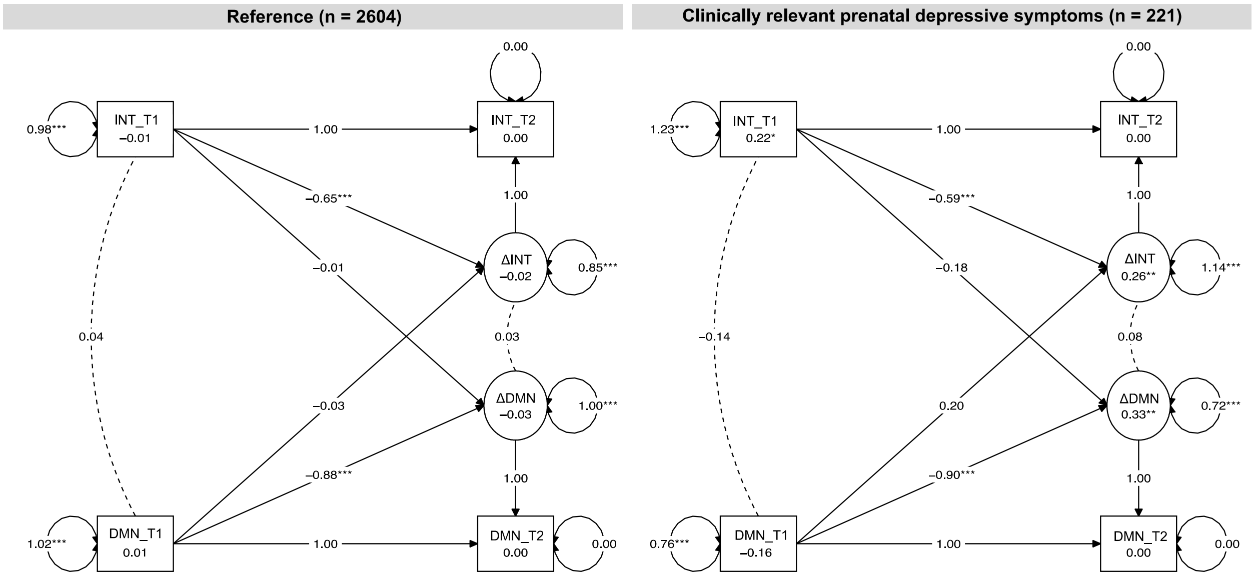

Note: Standardized effects from the BLCS model were presented. T1 represents the baseline, and T2 is the follow-up score. dINT and dDMN denote the change scores in INT and DMN between baseline and follow-up, respectively. The model was conducted within a multi-group framework, where the exposure status (reference vs. clinically relevant prenatal depressive symptoms) served as the grouping variable. Models were adjusted for child sex, child age at the neuroimaging assessment, maternal age at intake, maternal national origin, marital status, maternal education level, maternal substance use (tobacco, cannabis, alcohol), monthly household income, in-scanner head motion (mean framewise displacement), and the age difference between the behavioral and rsfMRI assessment.

* p < .05; ** p < .01; *** p < .001

© 2025 Koc D et al. *JAMA Network Open.*

eFigure 7. Graph Showing the Multigroup Bivariate Latent Change Score (BLCS) Model for Child-Reported Externalizing (EXT) Problems and Global Efficiency (GE) Relations

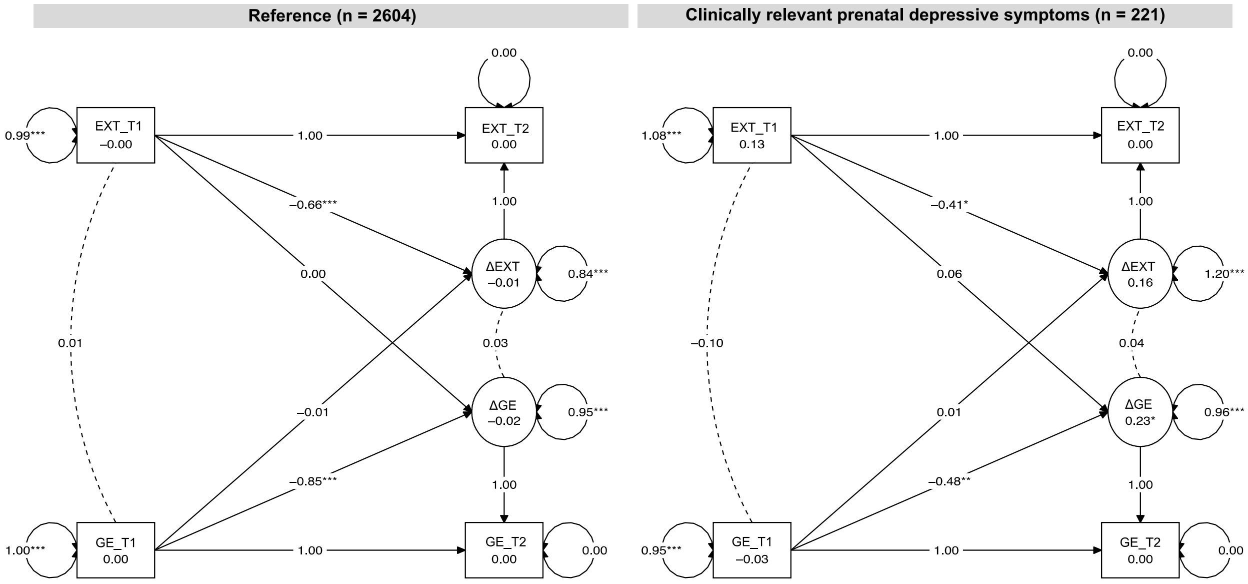

Note: Standardized effects from the BLCS model were presented. T1 represents the baseline, and T2 is the follow-up score. dEXT and dGE denote the change scores in EXT and GE between baseline and follow-up, respectively. The model was conducted within a multi-group framework, where the exposure status (reference vs. clinically relevant prenatal depressive symptoms) served as the grouping variable. Models were adjusted for child sex, child age at the neuroimaging assessment, maternal age at intake, maternal national origin, marital status, maternal education level, maternal substance use (tobacco, cannabis, alcohol), monthly household income, in-scanner head motion (mean framewise displacement), and the age difference between the behavioral and rsfMRI assessment.

* p < .05; ** p < .01; *** p < .001

© 2025 Koc D et al. *JAMA Network Open.*

eFigure 8. Graph Showing the Multigroup Bivariate Latent Change Score (BLCS) Model for Child-Reported Externalizing (EXT) Problems and Modularity (MOD) Relations

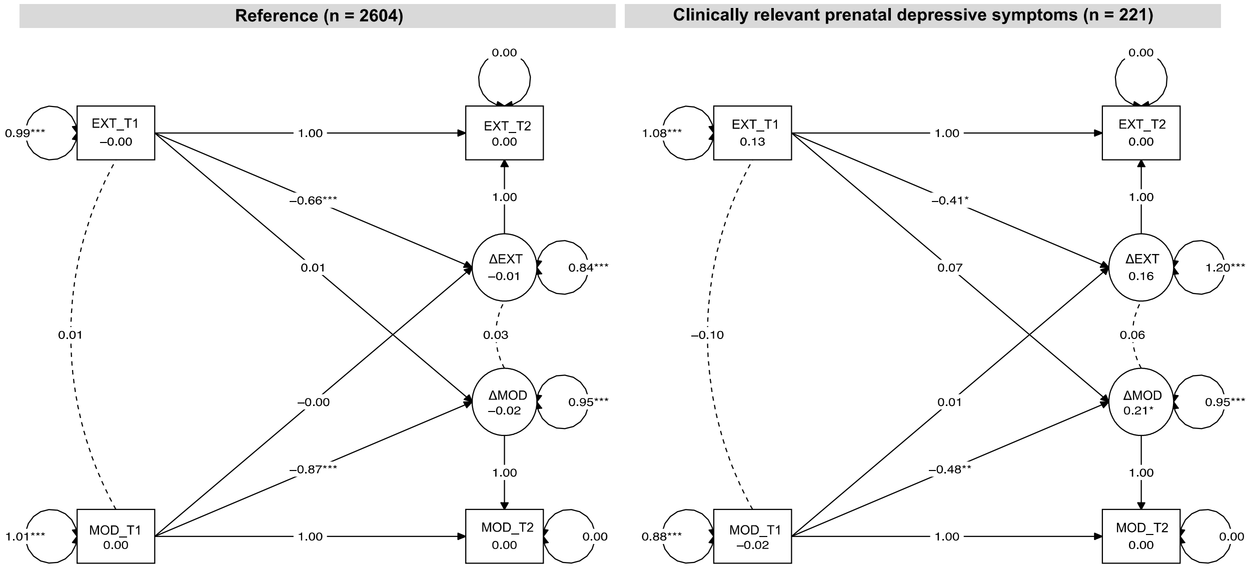

Note: Standardized effects from the BLCS model were presented. T1 represents the baseline, and T2 is the follow-up score. dEXT and dMOD denote the change scores in EXT and MOD between baseline and follow-up, respectively. The model was conducted within a multi-group framework, where the exposure status (reference vs. clinically relevant prenatal depressive symptoms) served as the grouping variable. Models were adjusted for child sex, child age at the neuroimaging assessment, maternal age at intake, maternal national origin, marital status, maternal education level, maternal substance use (tobacco, cannabis, alcohol), monthly household income, in-scanner head motion (mean framewise displacement), and the age difference between the behavioral and rsfMRI assessment.

* p < .05; ** p < .01; *** p < .001

© 2025 Koc D et al. *JAMA Network Open.*

eFigure 9. Graph Showing the Multigroup Bivariate Latent Change Score (BLCS) Model for Child-Reported Externalizing (EXT) Problems and Default Mode Network (DMN) Relations

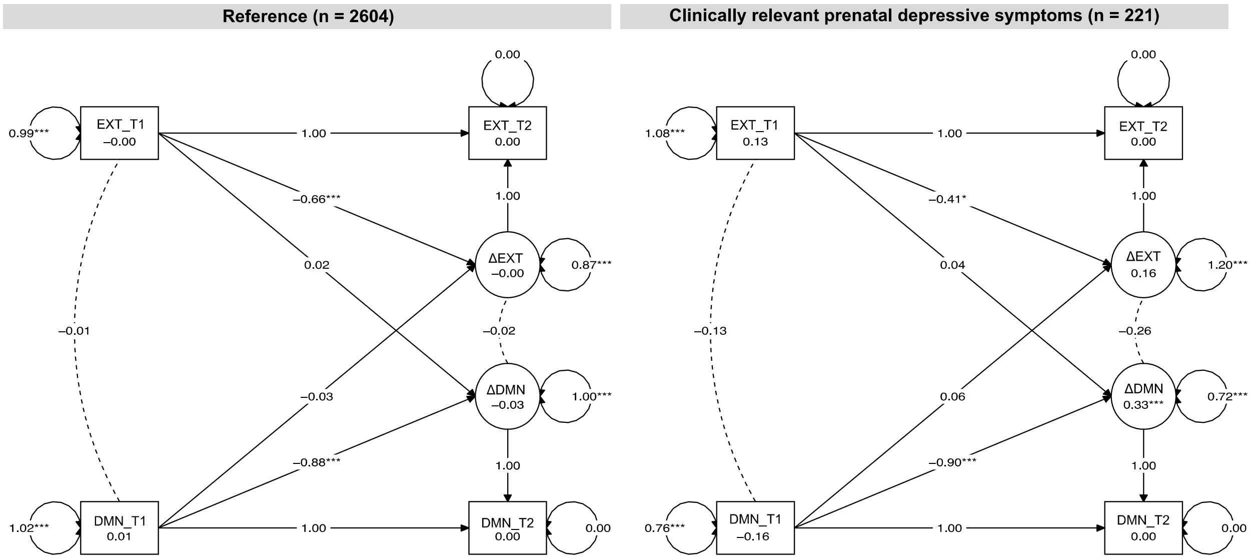

Note: Standardized effects from the BLCS model were presented. T1 represents the baseline, and T2 is the follow-up score. dEXT and dDMN denote the change scores in EXT and DMN between baseline and follow-up, respectively. The model was conducted within a multi-group framework, where the exposure status (reference vs. clinically relevant prenatal depressive symptoms) served as the grouping variable. Models were adjusted for child sex, child age at the neuroimaging assessment, maternal age at intake, maternal national origin, marital status, maternal education level, maternal substance use (tobacco, cannabis, alcohol), monthly household income, in-scanner head motion (mean framewise displacement), and the age difference between the behavioral and rsfMRI assessment.

* p < .05; ** p < .01; *** p < .001

© 2025 Koc D et al. *JAMA Network Open.*

eTable 1. Number of Images of the Participants in Each Group (Prenatal Depression vs Reference) at T1 and T2

**Time point**	Reference **(n = 2604)**	Clinically relevant prenatal depressive symptoms **(n = 221)**	Total number of images
T1	1820	139	1959
T2	1532	136	1668
All time points	3352	275	3627

Note: The bold numbers refer to unique participants, the non-bold numbers refer to the brain images. Reference group: a low score on depression symptoms during pregnancy; Prenatal depression: children exposed to clinically relevant depressive symptoms during pregnancy.

eTable 2. Number of Images of the Participants in Each Group (Postnatal Depression vs Reference) at T1 and T2

**Time point**	Reference **(n = 2102)**	Clinically relevant postnatal depressive symptoms **(n = 158)**	Total number of images
T1	1523	101	1624
T2	1207	92	1299
All time points	2730	193	2923

Note: The bold numbers refer to unique participants, the non-bold numbers refer to the brain images. Reference group: a low score on depression symptoms during the postnatal period; Postnatal depression: children exposed to clinically relevant depressive symptoms during the postnatal period.

© 2025 Koc D et al. *JAMA Network Open.*

eTable 3. Demographic Characteristics of the Study Population for Exposure Groups

	Total (n = 2825 with 3627 scans)		Total (n = 2260 with 2923 scans	
**N (%) or mean (S.D.)**	**Reference (n = 2604 with 3352 scans)**	**Clinically relevant prenatal depressive symptoms (n = 221 with 275 scans)**	**Reference (n = 2102 with 2730 scans)**	**Clinically relevant postnatal depressive symptoms (n = 158 with 193 scans)**
**Maternal characteristics**				
Maternal age at intake, years	31.2 (4.5)	28.4 (5.9)	31.6 (4.3)	29.6 (5.7)
Maternal national origin				
Dutch	1632 (62.7)	62 (28.1)	1394 (66.3)	61 (38.6)
Non-Dutch European	227 (8.7)	17 (7.7)	185 (8.8)	14 (8.9)
Non-European				
Caribbean	235 (9)	45 (20.4)	162 (7.7)	23 (14.6)
Moroccan/Turkish	215 (8.3)	53 (24)	134 (6.4)	35 (22.2)
African	122 (4.7)	35 (15.8)	83 (3.9)	15 (9.5)
Asian Oceanian	173 (6.6)	9 (4.1)	144 (6.9)	10 (6.3)
Marital status, with partner	2355 (90.4)	151 (68.3)	1925 (91.6)	122 (77.2)
Maternal education level				
Primary or lower	135 (5.2)	36 (16.3)	85 (4)	17 (10.8)
Secondary	1030 (39.6)	128 (57.9)	765 (36.4)	83 (52.5)
Higher	1439 (55.2)	57 (25.8)	1252 (59.6)	58 (36.7)
Monthly household income (€/month)				
<1200	377 (12.9)	108 (48.9)	221 (10.5)	59 (37.3)
1,200–2000	332 (12.7)	45 (20.4)	244 (11.6)	29 (18.4)
>2000	1935 (74.4)	68 (30.8)	1637 (77.9)	70 (44.3)
Maternal tobacco use				
Never during pregnancy	2021 (77.6)	139 62.9)	1656 (78.8)	105 (66.5)
Until pregnancy was known	233 (8.9)	20 (9)	202 (9.6)	18 (11.4)
Continued during pregnancy	350 (13.4)	62 (28.1)	244 (11.6)	35 (22.1)
Maternal cannabis use				

© 2025 Koc D et al. *JAMA Network Open.*

Never during pregnancy	2498 (94.9)	2237 (95.2)	2004 (95.3)	145 (91.8)
Before pregnancy	73 (2.8)	68 (2.9)	59 (2.8)	4 (2.5)
During pregnancy	61 (2.3)	46 (2)	39 (1.9)	9 (5.7)
Maternal alcohol use				
Never during pregnancy	945 (36.3)	99 (44.8)	707 (33.6)	66 (41.8)
Until pregnancy was known	368 (14.1)	28 (12.7)	291 (13.8)	26 (16.4)
Throughout pregnancy, occasionally	990 (38)	86 (38.9)	849 (40.4)	49 (31)
Throughout pregnancy, frequently	301 (11.6)	8 (3.6)	255 (12.1)	17 (10.8)
Used antidepressant in pregnancy %	17 (0.6)	7 (3.2)	14 (0.7)	6 (3.8)
Maternal depressive symptoms score [b]				
20 weeks of gestation	0.1 (0.1)	1.45 (0.6)	0.13 (0.2)	0.80 (0.6)
Child age 2 months (n = 2004)	0.13 (0.3)	0.87 (0.6)	0.1 (0.2)	1.4 (0.7)
Child age 6 months (n = 1802)	0.17 (0.3)	0.97 (0.8)	0.12 (0.2)	1.54 (1)
**Child characteristics**				
Child sex, male	1221 (46.9)	108 (48.9)	979 (46.6)	80 (50.6)
Gestational age at birth, week	39.9 (1.7)	39.7 (2.1)	39.9 (1.6)	39.6 (1.8)
Birth weight, grams	3472.5 (553.7)	3315.3 (582.7)	3484.1 (537.9)	3418.3 (521.7)
Child psychopathology (median, IQR)				
T1 (raw scores) (n =1577)				
Internalizing symptoms	2 (0-3)	2 (1-4)	2 (0-3)	2 (0-3)
Externalizing symptoms	2 (0-3)	2 (0-3)	2 (0-3)	2 (0-3.75)
T2 (raw scores) (n = 1443)				
Internalizing symptoms	7 (4-12)	10 (5-16)	7 (3.5-12)	8 (4-13)
Externalizing symptoms	6 (3-10)	7 (3-12)	6 (3-9.9)	7 (4-11)
Child age at neuroimaging, years, mean (SD), min-max				
T1	10.2 (0.6), 8.7-13	10.2 (0.7), 9.4-12.2	10.1 (0.6), 8.7-13	10.1 (0.6), 9.2-13
T2	13.9 (0.6), 12.6-17.1	14.1 (0.6), 12.8-16	13.9 (0.6), 12.9-17.1	13.9 (0.6), 12.9-16.5

[a] Pooled imputed data are shown (except for maternal depressive symptom and child psychopathology scores).

[b] Scores range from 0 to 4, with higher scores indicating higher depressive symptoms.

© 2025 Koc D et al. *JAMA Network Open.*

eTable 4. Non-Response Analysis

Characteristics	Responders[a] (n = 2825)	Non-responders (n= 3814)	P-value
Child sex, male (%)	47	52	<.001
Missing (%)	0	1.5	
Gestational age at birth, week	39.9 (1.7)	39.9 (1.8)	.19
Missing (%)	0.3	0.5	
Maternal national origin (%)			
Missing (%)	1.8	8.2	
Dutch	60	49.2	<.001
Non-Dutch European	8.6	9.3	
Non-European			
Caribbean	9.9	13	
Moroccan/Turkish	9.5	16.4	
African	5.6	7.1	
Asian Oceanian	6.4	5.1	
Marital status, with partner (%)	88.7	84.6	<.001
Missing (%)	5.1	15.2	
Education level			
Missing (%)	4.7	15.5	
Primary or lower	6	11.9	<.001
Secondary	41	49	
Higher	53	39.1	
Household income (euros/month) (%)			
Missing (%)	18.7	39.4	
<1200	15.8	28.3	<.001
1200–2000	13.3	16.5	
>2000	70.9	55.2	
Maternal tobacco use (%)			
Missing (%)	8.4	18.5	

© 2025 Koc D et al. *JAMA Network Open.*

Never during pregnancy	77.6	70.6	<.001
Until pregnancy was known	9.0	8.4	
Continued during pregnancy	13.3	20.9	
Maternal cannabis use (%)			
Missing (%)	10.2	18.7	
Never during pregnancy	94.9	93.1	.007
Before pregnancy	2.8	3.4	
During pregnancy	2.3	3.5	
Maternal alcohol use (%)			
Missing (%)	6.3	19.1	
Never during pregnancy	37.3	49.2	<.001
Until pregnancy was known	14.2	12.9	
Throughout pregnancy, occasionally	37.7	31.2	
Throughout pregnancy, frequently	10.8	6.8	
Used antidepressant in pregnancy %	0.9	1.5	<.001
Maternal age at intake, mean (SD) (years)	31.1 (4.7)	29.3 (5.3)	<.001
Missing (%)	0	0	
Maternal depressive symptom score [b] ,mean (SD)			
20 weeks of gestation	0.2 (0.4)	0.28 (0.54)	<.001
Child age 2 months (n = 2004 and 2022)	0.18 (0.42)	0.24 (0.5)	<.001
Child age 6 months (n = 1802 and 1678)	0.22 (0.48)	0.24 (0.5)	0.13

Note: Non-respondents are participants with data on maternal depression (20 weeks of gestation), but no at least useable neuroimaging data at follow-up. P-values were derived from t-tests or Wilcoxon tests for continuous variables and chi-square tests for categorical variables.

[a] Imputed data were reported (except for maternal depressive symptom scores).

[b] Scores range from 0 to 4, with higher scores indicating more clinically relevant psychological symptoms.

© 2025 Koc D et al. *JAMA Network Open.*

eTable 5. Comparison of Results From the Unweighted Model With Inverse Probability Of Attrition Weighting (IPAW) Models

	Unweighted model		IPAW-weighted model		
	**AIC**	**BIC**	**AIC**	**BIC**	**P-value**
**Brain-wide functional architecture**					
Global efficiency	-10580	-10433	-10526	-10379	< .001
Modularity	-10725	-10578	-10669	-10522	< .001
Clustering coefficient	-14421	-14274	-14378	-14231	< .001
**Within-Network RSFC**					
CON	-6173.4	-6029.5	-6095.2	-5951.4	< .001
DAN	-6327.0	-6183.1	-6255.6	-6111.8	< .001
DMN	-6707.3	-6563.4	-6669.1	-6525.2	< .001
FPN	-6770.1	-6697.2	-6697.2	-6553.4	< .001
PON	-5318.4	-5150.4	-5294.4	-5050.0	< .001
MPN	-6001.2	-5844.5	-5922.9	-5802.4	< .001
SAN	-3912.6	-3768.7	-3856.0	-3712.1	< .001
SMH	-5117.7	-4973.9	-5024.7	-4880.8	< .001
SMM	-3465.5	-3321.7	-3436.5	-3292.7	< .001
VAN	-6457.3	-6313.4	-6403.7	-6259.9	< .001
AUD	-4774.2	-4630.3	-4719.6	-4575.8	< .001
**Between-Network RSFC**					
CON	-8611.3	-8467.5	-8568.5	-8424.6	< .001
DAN	-8056.5	-7912.7	-7998.5	-7854.6	< .001
DMN	-8813.5	-8669.6	-8757.2	-8613.3	< .001
FPN	-8226.7	-8082.8	-8167.4	-8023.6	< .001
PON	-8326.4	-8150.0	-8234.1	-8050.3	< .001
MPN	-8633.1	-8453.6	-8611.4	-8421.4	< .001

© 2025 Koc D et al. *JAMA Network Open.*

SAN	-8487.6	-8343.8	-8439.9	-8296.0	< .001
SMH	-7086.6	-6942.7	-7032.7	-6888.8	< .001
SMM	-6750.7	-6606.8	-6678.5	-6524.6	< .001
VAN	-8166.9	-8023.0	-8121.1	-7977.3	< .001
AUD	-7896.3	-7752.4	-7825.8	-7682.0	< .001

Note: In all children (n=2825), unweighted models were compared to IPAW-weighted model models using the likelihood ratio test. A p-value < .05 suggests an improved model fit of the unweighted models. Models were adjusted for child sex and age at the neuroimaging assessment, maternal age at intake, maternal ethnicity, marital status, maternal education level, maternal substance use (tobacco, cannabis, alcohol) during pregnancy, monthly household income, and in-scanner head motion (mean framewise displacement). AIC = Akaike information criterion. BIC = Bayesian information criterion. AUD, auditory network; CON, cingulo-opercular network; DAN, dorsal attention network; DMN, default mode network; FPN, frontoparietal network; PON, parieto-occipital network; MPN, medial parietal network; RSFC, resting-state functional connectivity; SAN, salience network; SMH, sensorimotor hand network; SMM, sensorimotor mouth network; VAN, ventral attention network; VIS, visual network.

Network-related functional connectivity was characterized by two distinct measures: within-network connectivity, calculated as the mean connectivity between nodes within each network (eFigure 4A), and between-network connectivity, calculated as the mean connectivity between nodes in one network and nodes in all other networks (eFigure 4B).

© 2025 Koc D et al. *JAMA Network Open.*

eTable 6. Association of Clinically Relevant Maternal Depressive Symptoms With Offspring Brain-Wide Functional Architecture

	**Clinically relevant prenatal depressive symptoms (n =221) vs Reference (n=2604)**			**Clinically relevant postnatal depressive symptoms (n =158) vs Reference (n=2102)**		
	Estimate (SE)	$P_{\text{unadjusted}}$	P_{FDR}	Estimate (SE)	$P_{\text{unadjusted}}$	P_{FDR}
**Global efficiency**						
Main effect	0.005 (0.003)	.12	.21	0.010 (0.005)	.04	.06
Interaction effect	0.004 (0.001)	.004	**.006***	0.000 (0.002)	.91	.91
**Modularity**						
Main effect	0.004 (0.003)	.14	.21	0.011 (0.004)	.03	.06
Interaction effect	0.010 (0.003)	.001	**.003***	0.000 (0.004)	.90	.91
**Clustering coefficient**						
Main effect	0.002 (0.002)	.30	.30	-0.009 (0.007)	.37	.37
Interaction effect	-0.003 (0.002)	.10	.10	0.001 (0.002)	.85	.91

Note: Linear mixed-effect models were used to test the associations of prenatal and postnatal maternal depressive symptoms exposure and repeatedly assessed brain-wide functional architecture from age 9 to 15 years. Effect estimates including the main effect and interaction effect (β) (metric change; interaction of group*age), as well as standard errors (SE), $P_{\text{unadjusted}}$ and P_{FDR} values are shown. The main effect estimates the difference in functional connectivity between the exposure groups versus the reference. All models were adjusted for child sex and age at the neuroimaging assessment, maternal age at intake, maternal ethnicity, marital status, maternal education level, maternal substance use (tobacco, cannabis, alcohol), monthly household income, in-scanner head motion (mean framewise displacement), and prenatal depression scores was added for clinically relevant postnatal depressive symptoms model.

*Indicates significant associations after false discovery rate (FDR) correction for multiple testing for graph theory metrics.

© 2025 Koc D et al. *JAMA Network Open.*

eTable 7. Association of Maternal Depressive Symptoms (Continuous) With Offspring Within- and Between-Network Functional Connectivity.

Outcomes:	**Prenatal depressive symptoms (n =2825)**			**Postnatal depressive symptoms (n =2260)**		
**Within-Network RSFC**	Estimate (SE)	$P_{unadjusted}$	P_{FDR}	Estimate (SE)	$P_{unadjusted}$	P_{FDR}
**CON**						
Main effect	-0.016 (0.007)	.03	.48	-0.007 (0.014)	.27	.84
Interaction effect	0.004 (0.002)	.04	.19	0.002 (0.002)	.16	.54
**DAN**						
Main effect	-0.013 (0.008)	.04	.48	-0.001 (0.006)	.77	.84
Interaction effect	0.004 (0.002)	.03	.19	0.003 (0.002)	.10	.48
**DMN**						
Main effect	0.001 (0.003)	.67	.77	0.020 (0.013)	.06	.48
Interaction effect	0.010 (0.001)	< .001	**.002***	0.003 (0.002)	.05	.30
**FPN**						
Main effect	-0.009 (0.005)	.09	.48	-0.009 (0.005)	.09	.54
Interaction effect	0.003 (0.001)	.04	.19	0.003 (0.001)	.04	.30
**PON**						
Main effect	0.001 (0.009)	.86	.93	-0.011 (0.010)	.28	.84
Interaction effect	0.002 (0.004)	.57	.65	0.010 (0.005)	.04	.30
**MPN**						
Main effect	-0.004 (0.011)	.68	.77	-0.041 (0.050)	.48	.84
Interaction effect	-0.000 (0.004)	.94	.96	0.001 (0.005)	.71	.75
**SAN**						
Main effect	0.006 (0.009)	.94	.94	0.007 (0.009)	.44	.84
Interaction effect	-0.000 (0.002)	.95	.96	-0.000 (0.003)	.82	.82
**SMH**						

Main effect	-0.004 (0.007)	.54	.68	-0.005 (0.007)	.49	.84
Interaction effect	0.002 (0.002)	.24	.40	0.003 (0.002)	.18	.54
**SMM**						
Main effect	-0.011 (0.009)	.24	.54	-0.010 (0.010)	.28	.84
Interaction effect	0.003 (0.002)	.26	.40	0.006 (0.003)	.05	.30
**VAN**						
Main effect	-0.008 (0.005)	.16	.54	0.006 (0.003)	.04	.48
Interaction effect	0.003 (0.001)	.08	.27	0.001 (0.001)	.54	.64
**AUD**						
Main effect	-0.008 (0.007)	.28	.54	-0.001 (0.008)	.84	.84
Interaction effect	0.005 (0.002)	.04	.19	0.003 (0.002)	.13	.52
**VIS**						
Main effect	-0.001 (0.008)	.90	.94	0.012 (0.008)	.04	.48
Interaction effect	-0.000 (0.002)	.96	.96	-0.001 (0.002)	.72	.75
**Between-Network RSFC**	Estimate (SE)	$P_{\text{unadjusted}}$	P_{FDR}	Estimate (SE)	$P_{\text{unadjusted}}$	P_{FDR}
**CON**						
Main effect	-0.017 (0.015)	.24	.54	-0.010 (0.016)	.53	.84
Interaction effect	0.001 (0.001)	.16	.38	0.001 (0.001)	.32	.64
**DAN**						
Main effect	-0.027 (0.016)	.10	.48	-0.011 (0.017)	.55	.84
Interaction effect	0.002 (0.001)	.08	.27	0.001 (0.001)	.30	.64
**DMN**						
Main effect	-0.015 (0.014)	.30	.54	-0.003 (0.015)	.82	.84
Interaction effect	0.001 (0.001)	.27	.40	0.000 (0.001)	.49	.64
**FPN**						
Main effect	-0.015 (0.016)	.34	.54	-0.005 (0.017)	.74	.84
Interaction effect	0.001 (0.001)	.26	.40	0.001 (0.001)	.45	.64
**PON**						

© 2025 Koc D et al. *JAMA Network Open.*

Main effect	-0.032 (0.027)	.24	.54	0.006 (0.040)	.75	.84
Interaction effect	0.003 (0.002)	.18	.39	0.000 (0.002)	.72	.75
**MPN**						
Main effect	-0.015 (0.024)	.52	.68	0.014 (0.035)	.41	.84
Interaction effect	0.001 (0.002)	.45	.54	-0.000 (0.002)	.43	.64
**SAN**						
Main effect	-0.021 (0.015)	.15	.54	-0.011 (0.016)	.48	.84
Interaction effect	0.002 (0.001)	.13	.34	0.001 (0.001)	.24	.57
**SMH**						
Main effect	-0.012 (0.019)	.50	.68	-0.015 (0.021)	.46	.84
Interaction effect	0.001 (0.001)	.43	.54	0.002 (0.001)	.23	.64
**SMM**						
Main effect	-0.016 (0.020)	.41	.61	-0.012 (0.022)	.57	.84
Interaction effect	0.001 (0.001)	.37	.49	0.001 (0.001)	.35	.64
**VAN**						
Main effect	-0.015 (0.016)	.34	.54	-0.005 (0.017)	.76	84
Interaction effect	0.001 (0.001)	.29	.40	0.001 (0.001)	.49	.64
**AUD**						
Main effect	-0.027 (0.017)	.10	.48	-0.007 (0.018)	.66	.84
Interaction effect	0.002 (0.001)	.09	.27	0.001 (0.003)	.44	.64
**VIS**						
Main effect	-0.020 (0.017)	.25	.54	-0.006 (0.019)	.72	.84
Interaction effect	0.001 (0.001)	.25	.40	0.001 (0.001)	.53	.64

Note: Linear mixed-effect models were used to test the associations of prenatal and postnatal maternal depressive symptoms (continuous) exposure with repeatedly assessed functional network connectivity from ages 9 to 15 years. Effect estimates including the main effect and interaction effect (β) (functional connectivity change; interaction of depressive symptoms score*age), as well as standard errors (SE), Unadjusted and FDR corrected values are shown. All models were adjusted for child sex, child age at the

© 2025 Koc D et al. *JAMA Network Open.*

neuroimaging assessment, maternal age at intake, maternal national origin, marital status, maternal education level, maternal substance use (tobacco, cannabis, alcohol), monthly household income, and in-scanner head motion (mean framewise displacement). The postnatal depressive symptoms model was additionally adjusted for prenatal depression scores. AUD, auditory network; CON, cingulo-opercular network; DAN, dorsal attention network; DMN, default mode network; FPN, frontoparietal network; PON, parieto-occipital network; MPN, medial parietal network; RSFC, resting-state functional connectivity; SAN, salience network; SMH, sensorimotor hand network; SMM, sensorimotor mouth network; VAN, ventral attention network; VIS, visual network.

Network-related functional connectivity was characterized by two distinct measures: within-network connectivity, calculated as the mean connectivity between nodes within each network (eFigure 4A), and between-network connectivity, calculated as the mean connectivity between nodes in one network and nodes in all other networks (eFigure 4B).

*****Indicates significant associations after false discovery rate (FDR) correction for multiple testing for RSFC networks.

© 2025 Koc D et al. *JAMA Network Open.*

eTable 8. Association of Clinically Relevant Maternal Depressive Symptoms With Offspring Within- and Between-Network Functional Connectivity Using the Gordon Parcellation.

	**Clinically relevant prenatal depressive symptoms (n =221) vs Reference (n=2604)**			**Clinically relevant postnatal depressive symptoms (n =158) vs Reference (n=2102)**		
**Within-Network RSFC**	Estimate (SE)	$P_{\text{unadjusted}}$	P_{FDR}	Estimate (SE)	$P_{\text{unadjusted}}$	P_{FDR}
**CON**						
Main effect	-0.023 (0.011)	.03	.24	0.012 (0.014)	.40	.99
Interaction effect	0.007 (0.003)	.04	.24	0.002 (0.004)	.55	.88
**DAN**						
Main effect	-0.013 (0.010)	.19	.54	0.013 (0.014)	.32	.99
Interaction effect	0.006 (0.003)	.03	.24	0.001 (0.004)	.73	.88
**DMN**						
Main effect	0.021 (0.010)	.02	.24	0.005 (0.013)	.96	.99
Interaction effect	0.011 (0.003)	< .001	**.002***	0.002 (0.004)	.50	.88
**FPN**						
Main effect	-0.073 (0.034)	.03	.24	-0.022 (0.040)	.65	.99
Interaction effect	0.007 (0.005)	.32	.40	0.003 (0.004)	.47	.88
**PON**						
Main effect	-0.010 (0.012)	.20	.54	0.015 (0.012)	.37	.99
Interaction effect	0.005 (0.004)	.10	.36	0.001 (0.005)	.60	.88
**MPN**						
Main effect	-0.015 (0.011)	.22	.54	0.020 (0.014)	.37	.99
Interaction effect	0.006 (0.004)	.15	.36	0.002 (0.004)	.50	.88
**SAN**						
Main effect	-0.005 (0.015)	.71	.74	0.032 (0.020)	.44	.99
Interaction effect	0.004 (0.004)	.37	.43	-0.003 (0.006)	.65	.88

© 2025 Koc D et al. *JAMA Network Open.*

**SMH**						
Main effect	-0.054 (0.045)	.22	.54	0.001 (0.017)	.22	.99
Interaction effect	0.006 (0.004)	.12	.36	0.004 (0.005)	.40	.88
**SMM**						
Main effect	-0.003 (0.016)	.83	.83	0.025 (0.022)	.26	.99
Interaction effect	0.006 (0.004)	.24	.36	0.002 (0.007)	.77	.88
**VAN**						
Main effect	-0.008 (0.009)	.38	.57	0.028 (0.013)	.03	.48
Interaction effect	0.005 (0.003)	.05	.24	-0.002 (0.004)	.52	.88
**AUD**						
Main effect	-0.013 (0.014)	.30	.54	0.011 (0.018)	.51	.99
Interaction effect	0.007 (0.004)	.05	.24	0.005 (0.006)	.40	.88
**VIS**						
Main effect	0.007 (0.013)	.55	.63	0.038 (0.018)	.04	.48
Interaction effect	0.000 (0.004)	.90	.90	-0.006 (0.006)	.27	.88
**Between-Network RSFC**	Estimate (SE)	$P_{unadjusted}$	P_{FDR}	Estimate (SE)	$P_{unadjusted}$	P_{FDR}
**CON**						
Main effect	-0.026 (0.020)	.30	.54	-0.001 (0.036)	.96	.99
Interaction effect	0.002 (0.003)	.20	.36	0.001 (0.003)	.73	.88
**DAN**						
Main effect	-0.032 (0.027)	.24	.54	0.006 (0.040)	.87	.99
Interaction effect	0.003 (0.002)	.18	.36	0.000 (0.003)	.85	.92
**DMN**						
Main effect	-0.015 (0.024)	.52	.62	0.014 (0.035)	.67	.99
Interaction effect	0.001 (0.002)	.45	.49	0.000 (0.003)	.98	.98
**FPN**						
Main effect	-0.020 (0.026)	.43	.57	-0.003 (0.038)	.90	.99

© 2025 Koc D et al. *JAMA Network Open.*

Interaction effect	0.002 (0.003)	.30	.40	0.001 (0.003)	.64	.88
**PON**						
Main effect	-0.025 (0.021)	.28	.54	0.005 (0.037)	.85	.99
Interaction effect	0.002 (0.002)	.19	.36	0.001 (0.003)	.70	.88
**MPN**						
Main effect	-0.030 (0.023)	.31	.54	0.008 (0.038)	.82	.99
Interaction effect	0.003 (0.002)	.21	.36	0.001 (0.003)	.75	.88
**SAN**						
Main effect	-0.026 (0.025)	.31	.54	0.001 (0.037)	.96	.99
Interaction effect	0.002 (0.002)	.23	.36	0.001 (0.003)	.71	.88
**SMH**						
Main effect	-0.031 (0.032)	.33	.54	-0.010 (0.047)	.83	.99
Interaction effect	0.003 (0.002)	.21	.36	0.002 (0.004)	.52	.88
**SMM**						
Main effect	0.018 (0.034)	.59	.64	-0.007 (0.049)	.88	.99
Interaction effect	0.001 (0.002)	.54	.56	0.001 (0.004)	.67	.88
**VAN**						
Main effect	-0.020 (0.027)	.44	.57	-0.008 (0.039)	.83	.99
Interaction effect	0.002 (0.002)	.32	.40	0.001 (0.003)	.61	.88
**AUD**						
Main effect	-0.037 (0.02)	.18	.54	0.000 (0.040)	.99	.99
Interaction effect	0.003 (0.002)	.12	.36	0.001 (0.003)	.77	.88
**VIS**						
Main effect	-0.022 (0.029)	.45	.57	0.008 (0.030)	.84	.99
Interaction effect	0.002 (0.003)	.38	.43	0.000 (0.003)	.97	.88

Note: Linear mixed-effect models were used to test the associations of prenatal and postnatal maternal depressive symptoms (exposure and repeatedly assessed functional network connectivity from age 9 to 15 years. Effect

© 2025 Koc D et al. *JAMA Network Open.*

estimates including the main effect and interaction effect (β) (functional connectivity change; interaction of group*age), as well as standard errors (SE), $P_{unadjusted}$ and P_{FDR} values are shown. The main effect estimates the difference in functional connectivity between the exposure groups versus the reference. All models were adjusted for child sex and age at the neuroimaging assessment, maternal age at intake, maternal ethnicity, marital status, maternal education level, maternal substance use (tobacco, cannabis, alcohol), monthly household income, and in-scanner head motion (mean framewise displacement), and prenatal depression scores was added for clinically relevant postnatal depressive symptoms model. AUD, auditory network; CON, cingulo-opercular network; DAN, dorsal attention network; DMN, default mode network; FPN, frontoparietal network; PON, parieto-occipital network; MPN, medial parietal network; RSFC, resting-state functional connectivity; SAN, salience network; SMH, sensorimotor hand network; SMM, sensorimotor mouth network; VAN, ventral attention network; VIS, visual network.

Network-related functional connectivity was characterized by two distinct measures: within-network connectivity, calculated as the mean connectivity between nodes within each network (eFigure 4A), and between-network connectivity, calculated as the mean connectivity between nodes in one network and nodes in all other networks (eFigure 4B).

*Indicates significant associations after false discovery rate (FDR) correction for multiple testing for RSFC networks.

© 2025 Koc D et al. *JAMA Network Open.*

eTable 9. Association of Clinically Relevant Maternal Depressive Symptoms With Offspring Brain-Wide Functional Architecture (Graph Theory Measures)

	**Clinically relevant prenatal depressive symptoms (n =221) vs Reference (n=2604)**			**Clinically relevant postnatal depressive symptoms (n =158) vs Reference (n=2102)**		
	Statistic	$P_{unadjusted}$	P_{FDR}	Statistic	$P_{unadjusted}$	P_{FDR}
**Global efficiency**						
Group	t = 1.5	.13	.19	t = 1.4	.14	.17
s(age):Group	*F* = 14.5	< .001	**< .001***	*F* = 2.3	.02	.06
**Modularity**						
Group	t = 1.6	.11	.19	t = 1.7	.09	.17
s(age):Group	*F* = 8.1	< .001	**< .001***	*F* = 2.1	.04	.06
**Clustering coefficient**						
Group	t = 0.72	.46	.46	t = -1.36	.17	.17
s(age):Group	*F* = 0.1	.67	.67	*F* = 0.9	.26	.31

Note: Generalized additive mixed models were used to test the associations of prenatal and postnatal maternal depressive symptoms exposure and repeatedly assessed brain-wide functional architecture from age 9 to 15 years. Statistics are reported as t statistics for parametric terms (main effect) and F statistics for smooth terms (interaction effect, s(age):Group). Age was modeled as a smooth term, allowing for non-linear effects and group-specific trajectories. Models were additionally adjusted for child sex, maternal age at intake, maternal ethnicity, marital status, maternal education level, maternal substance use (tobacco, cannabis, alcohol), monthly household income, in-scanner head motion (mean framewise displacement), and prenatal depression scores was added for clinically relevant postnatal depressive symptoms model. *Indicates significant associations after false discovery rate correction (FDR) for multiple testing for global graph metrics.

© 2025 Koc D et al. *JAMA Network Open.*

eTable 10. Association of Clinically Relevant Maternal Depressive Symptoms With Offspring Within- and Between-Network Functional Connectivity Using the Gordon Parcellation

	**Clinically relevant prenatal depressive symptoms (n =221) vs Reference (n=2604)**			**Clinically relevant postnatal depressive symptoms (n =158) vs Reference (n=2102)**		
**Within-Network RSFC**	Statistic	$P_{unadjusted}$	P_{FDR}	Statistic	$P_{unadjusted}$	P_{FDR}
**CON**						
Group	t = -1.2	.25	.42	t = 2.1	.05	.26
s(age):Group	*F* = 2.3	.02	.40	*F* = 2.4	.18	.44
**DAN**						
Group	t = -0.52	.60	.68	t = 1.9	.05	.26
s(age):Group	*F* = 1.6	.11	.53	*F* = 1.2	.35	.69
**DMN**						
Group	t = 2.1	.03	.42	t = 0.86	.38	.69
s(age):Group	*F* = 18.6	< .001	**.004***	*F* = 2.9	.08	.26
**FPN**						
Group	t = -1.3	.16	.42	t = -1.4	.17	.44
s(age):Group	*F* = 0.9	.41	.61	*F* = 0.2	.74	.87
**PON**						
Group	t = -1.0	.20	.42	t = 1.5	.07	.30
s(age):Group	*F* = 1.5	.18	.50	*F* = 0.9	.32	.60
**MPN**						
Group	t = -1.2	.22	.42	t = 1.6	.08	.32
s(age):Group	*F* = 1.6	.16	.50	*F* = 1.1	.30	.57
**SAN**						
Group	t = -0.2	.81	.81	t = 1.8	.06	.26
s(age):Group	*F* = 2.9	.08	.53	*F* = 0.03	.85	.91
**SMH**						

© 2025 Koc D et al. *JAMA Network Open.*

Group	t = -1.2	.06	.42	t =-0.7	.50	.78
s(age):Group	*F* = 1.1	.36	.61	*F* = 0.8	.43	.71
**SMM**						
Group	t = -0.2	.70	.73	t = 1.4	.09	.27
s(age):Group	*F* = 0.5	.51	.63	*F* = 0.2	.69	.86
**VAN**						
Group	t = -0.7	.43	.60	t = 2.5	.01	.26
s(age):Group	*F* = 1.1	.42	.61	*F* = 0.5	.52	.69
**AUD**						
Group	t = -0.5	.60	.68	t = 2.1	.03	.26
s(age):Group	*F* = 3.1	.13	.53	*F* = 0.9	.40	.69
**VIS**						
Group	t = 1.1	.29	.46	t = 1.8	.06	.26
s(age):Group	*F* = 0.7	.46	.61	*F* = 1.1	.36	.69
**Between-Network RSFC**	Statistic	$P_{unadjusted}$	P_{FDR}	Statistic	$P_{unadjusted}$	P_{FDR}
**CON**						
Group	t = -1.3	.49	.65	t = -0.01	.94	.98
s(age):Group	*F* = 1.5	.27	.59	*F* = 0.04	.85	.91
**DAN**						
Group	t = -1.1	.22	.42	t = 0.2	.85	.91
s(age):Group	*F* = 1.2	.45	.61	*F* = 0.001	.98	98
**DMN**						
Group	t = -1.2	.25	.42	t = -1.9	.05	.26
s(age):Group	*F* = 0.7	.57	.64	*F* = 0.6	.57	.78
**FPN**						
Group	t = -1.4	.14	.42	t = -1.8	.06	.26
s(age):Group	*F* = 1.6	.30	.59	*F* = 0.7	.54	.78
**PON**						
Group	t = -1.0	.21	.42	t = 1.6	.07	.30

© 2025 Koc D et al. *JAMA Network Open.*

s(age):Group	*F* = 1.2	.33	.59	*F* = 0.5	.52	.78
**MPN**						
Group	t = -1.2	.23	.42	t = 1.5	.08	.32
s(age):Group	*F* = 1.3	.30	.59	*F* = 0.6	.50	.77
**SAN**						
Group	t = -1.1	.31	.42	t = 0.04	.96	.98
s(age):Group	*F* = 0.7	.45	.61	*F* = 0.32	.62	.80
**SMH**						
Group	t = -1.4	.15	.42	t = -1.8	.07	.26
s(age):Group	*F* = 0.4	.54	.64	*F* = 0.3	.59	.78
**SMM**						
Group	t = 0.3	.70	.73	t = -1.6	.10	.28
s(age):Group	*F* = 0.8	.42	.61	*F* = 0.4	.59	.78
**VAN**						
Group	t = -1.2	.21	.42	t = -1.7	.07	.26
s(age):Group	*F* = 1.7	.08	.53	*F* = 0.93	.40	.69
**AUD**						
Group	t = -1.2	.20	.42	t = 1.7	.08	.26
s(age):Group	*F* = 2.4	.13	.53	*F* = 1.7	.25	.55
**VIS**						
Group	t = -0.5	.60	.68	t = 1.3	.19	.44
s(age):Group	*F* = 0.9	.39	.61	*F* = 0.16	.72	.87

Note: Generalized additive mixed models were used to test the associations of prenatal and postnatal maternal depressive symptoms exposure and repeatedly assessed offspring within- and between-network functional connectivity using the Gordon parcellation from age 9 to 15 years. Statistics are reported as t statistics for parametric terms (main effect) and F statistics for smooth terms (interaction effect, s(age):Group). Age was modeled as a smooth term, allowing for non-linear effects and group-specific trajectories. Models were additionally adjusted for child sex, maternal age at intake, maternal ethnicity, marital status, maternal education

© 2025 Koc D et al. *JAMA Network Open.*

level, maternal substance use (tobacco, cannabis, alcohol), monthly household income, in-scanner head motion (mean framewise displacement), and prenatal depression scores was added for clinically relevant postnatal depressive symptoms model. AUD, auditory network; CON, cingulo-opercular network; DAN, dorsal attention network; DMN, default mode network; FPN, frontoparietal network; PON, parieto-occipital network; MPN, medial parietal network; RSFC, resting-state functional connectivity; SAN, salience network; SMH, sensorimotor hand network; SMM, sensorimotor mouth network; VAN, ventral attention network; VIS, visual network.

Network-related functional connectivity was characterized by two distinct measures: within-network connectivity, calculated as the mean connectivity between nodes within each network (eFigure 4A), and between-network connectivity, calculated as the mean connectivity between nodes in one network and nodes in all other networks (eFigure 4B).

*Indicates significant associations after false discovery rate correction (FDR) for multiple testing for RSFC networks.

© 2025 Koc D et al. *JAMA Network Open.*

eTable 11. Multigroup BLCS Model for Each Brain rsfMRI Metric.

	Reference (n = 2604)				Clinically relevant prenatal depressive symptoms (n = 221)			
	Estimate	SE	$P_{unadjusted}$	P_{FDR}	Estimate	SE	$P_{unadjusted}$	P_{FDR}
**Global efficiency**								
$INT_{t1} \leftrightarrow GE_{t1}$	0.003	0.027	.89	.97	-0.106	0.089	.23	.39
$INT_{t1} \rightarrow \Delta GE$	-0.048	0.030	.06	.36	0.243	0.37	<.001	**.001***
$GE_{t1} \rightarrow \Delta INT$	-0.036	0.022	.09	.36	0.047	0.078	.48	.57
$\Delta INT \leftrightarrow \Delta GE$	0.014	0.027	.62	.82	0.077	0.108	.47	.57
**Modularity**								
$INT_{t1} \leftrightarrow MOD_{t1}$	0.000	0.027	.99	.99	-0.073	0.095	.43	.57
$INT_{t1} \rightarrow \Delta MOD$	-0.047	0.020	.06	.36	0.205	0.113	.07	.28
$MOD_{t1} \rightarrow \Delta INT$	-0.032	0.022	.30	.51	0.036	0.078	.68	.68
$\Delta INT \leftrightarrow \Delta MOD$	0.021	0.027	.44	.66	0.136	0.107	.21	.39
**Default mode network**								
$INT_{t1} \leftrightarrow DMN_{t1}$	0.036	0.024	.13	.39	-0.144	0.104	.15	.39
$INT_{t1} \rightarrow \Delta DMN$	-0.005	0.025	.82	.97	-0.177	0.093	.06	.28
$DMN_{t1} \rightarrow \Delta INT$	-0.027	0.021	.21	.50	0.197	0.146	.18	.39
$\Delta INT \leftrightarrow \Delta DMN$	0.033	0.037	.30	.51	0.084	0.177	.62	.67

Note: Table showing results from the multigroup BLCS model of internalizing (INT) problems and rsfMRI measures of global efficiency (GE), modularity (MOD) and within default mode network (DMN) with estimates, standard error (SE), standard errors (SE), $P_{unadjusted}$ and P_{FDR} values. For each standardized effect, T1 represents the baseline score, dINT, dGE, dMOD, and dDMN represents the delta score between baseline and follow-up. Models were run within a multi-group framework, where exposure status (reference vs clinically relevant prenatal depressive symptoms)

© 2025 Koc D et al. *JAMA Network Open.*

was the grouping variable. Models were adjusted for child sex, child age at the neuroimaging assessment, maternal age at intake, maternal national origin, marital status, maternal education level, maternal substance use (tobacco, cannabis, alcohol), monthly household income, in-scanner head motion (mean framewise displacement), and age difference in behavioral and rsfMRI assessment. Model fit was good (Comparative fit index = 0.96, Root mean square error of approximation = 0.04).

***** Indicates significant associations after false discovery rate (FDR) correction for multiple testing

© 2025 Koc D et al. *JAMA Network Open.*

eReferences.

1. de Beurs, E. *Brief Symptom Inventory Handleiding (Brief Symptom Inventory Manual)*. PITS; 2004.

2. Marroun HE, White TJH, Knaap NJF van der, et al. Prenatal exposure to selective serotonin reuptake inhibitors and social responsiveness symptoms of autism: population-based study of young children. *The British Journal of Psychiatry*. 2014;205(2):95-102. doi:10.1192/bjp.bp.113.127746

3. Andrews G, Peters L. The psychometric properties of the Composite International Diagnostic Interview. *Soc Psychiatry Psychiatr Epidemiol*. 1998;33(2):80-88. doi:10.1007/s001270050026

4. Pop VJ, Komproe IH, van Son MJ. Characteristics of the Edinburgh post natal depression scale in The Netherlands. *Journal of Affective Disorders*. 1992;26(2):105-110. doi:10.1016/0165-0327(92)90041-4

5. El Marroun H, Jaddoe VWV, Hudziak JJ, et al. Maternal Use of Selective Serotonin Reuptake Inhibitors, Fetal Growth, and Risk of Adverse Birth Outcomes. *Archives of General Psychiatry*. 2012;69(7):706-714. doi:10.1001/archgenpsychiatry.2011.2333

6. Koc D, Tiemeier H, Stricker BH, Muetzel RL, Hillegers M, El Marroun H. Prenatal Antidepressant Exposure and Offspring Brain Morphologic Trajectory. *JAMA Psychiatry*. Published online August 30, 2023. doi:10.1001/jamapsychiatry.2023.3161

7. Koc D, El Marroun H, Stricker BH, Muetzel RL, Tiemeier H. Intrauterine Exposure to Antidepressants or Maternal Depressive Symptoms and Offspring Brain White Matter Trajectories From Late Childhood to Adolescence. *Biological Psychiatry: Cognitive Neuroscience and Neuroimaging*. Published online November 4, 2023. doi:10.1016/j.bpsc.2023.10.009

8. Achenbach TM, Rescorla LA. The Achenbach System of Empirically Based Assessment (ASEBA) for Ages 1.5 to 18 Years. In: *The Use of Psychological Testing for Treatment Planning and Outcomes Assessment: Instruments for Children and Adolescents, Volume 2, 3rd Ed*. Lawrence Erlbaum Associates Publishers; 2004:179-213.

9. White T, Muetzel RL, El Marroun H, et al. Paediatric population neuroimaging and the Generation R Study: the second wave. *Eur J Epidemiol*. 2018;33(1):99-125. doi:10.1007/s10654-017-0319-y

10. Ciric R, Wolf DH, Power JD, et al. Benchmarking of participant-level confound regression strategies for the control of motion artifact in studies of functional connectivity. *NeuroImage*. 2017;154:174-187. doi:10.1016/j.neuroimage.2017.03.020

© 2025 Koc D et al. *JAMA Network Open.*

11. Murphy K, Fox MD. Towards a consensus regarding global signal regression for resting state functional connectivity MRI. *NeuroImage*. 2017;154:169-173. doi:10.1016/j.neuroimage.2016.11.052

12. López-Vicente M, Agcaoglu O, Pérez-Crespo L, et al. Developmental Changes in Dynamic Functional Connectivity From Childhood Into Adolescence. *Frontiers in Systems Neuroscience*. 2021;15. Accessed December 6, 2023. https://www.frontiersin.org/articles/10.3389/fnsys.2021.724805

13. Farahani FV, Karwowski W, Lighthall NR. Application of Graph Theory for Identifying Connectivity Patterns in Human Brain Networks: A Systematic Review. *Frontiers in Neuroscience*. 2019;13. Accessed January 31, 2024. https://www.frontiersin.org/articles/10.3389/fnins.2019.00585

14. Cao M, Huang H, Peng Y, Dong Q, He Y. Toward Developmental Connectomics of the Human Brain. *Frontiers in Neuroanatomy*. 2016;10. Accessed March 1, 2024. https://www.frontiersin.org/articles/10.3389/fnana.2016.00025

15. Bullmore E, Sporns O. The economy of brain network organization. *Nat Rev Neurosci*. 2012;13(5):336-349. doi:10.1038/nrn3214

16. Rubinov M, Sporns O. Complex network measures of brain connectivity: Uses and interpretations. *NeuroImage*. 2010;52(3):1059-1069. doi:10.1016/j.neuroimage.2009.10.003

17. Bullmore E, Sporns O. Complex brain networks: graph theoretical analysis of structural and functional systems. *Nat Rev Neurosci*. 2009;10(3):186-198. doi:10.1038/nrn2575

18. Wood SN. Fast stable restricted maximum likelihood and marginal likelihood estimation of semiparametric generalized linear models. *Journal of the Royal Statistical Society: Series B (Statistical Methodology)*. 2011;73(1):3-36. doi:10.1111/j.1467-9868.2010.00749.x

19. Kievit RA, Brandmaier AM, Ziegler G, et al. Developmental cognitive neuroscience using latent change score models: A tutorial and applications. *Developmental Cognitive Neuroscience*. 2018;33:99-117. doi:10.1016/j.dcn.2017.11.007

20. Satorra A, Bentler PM. A scaled difference chi-square test statistic for moment structure analysis. *Psychometrika*. 2001;66(4):507-514. doi:10.1007/BF02296192

21. Cole SR, Hernán MA. Constructing Inverse Probability Weights for Marginal Structural Models. *American Journal of Epidemiology*. 2008;168(6):656-664. doi:10.1093/aje/kwn164

22. Lugo-Candelas C, Cha J, Hong S, et al. Associations Between Brain Structure and Connectivity in Infants and Exposure to Selective Serotonin Reuptake Inhibitors During Pregnancy. *JAMA Pediatr*. 2018;172(6):525-533. doi:10.1001/jamapediatrics.2017.5227

23. Hicks LM, Swales DA, Garcia SE, Driver C, Davis EP. Does Prenatal Maternal Distress Contribute to Sex Differences in Child Psychopathology? *Curr Psychiatry Rep*. 2019;21(2):7. doi:10.1007/s11920-019-0992-5

© 2025 Koc D et al. *JAMA Network Open.*

24. Buuren S van, Groothuis-Oudshoorn K. mice: Multivariate Imputation by Chained Equations in R. *Journal of Statistical Software*. 2011;45:1-67. doi:10.18637/jss.v045.i03

25. Multiple Imputation for Nonresponse in Surveys | Wiley Series in Probability and Statistics. Accessed December 24, 2024. https://onlinelibrary.wiley.com/doi/book/10.1002/9780470316696

26. Benjamini Y, Hochberg Y. Controlling the False Discovery Rate: A Practical and Powerful Approach to Multiple Testing. *Journal of the Royal Statistical Society: Series B (Methodological)*. 1995;57(1):289-300. doi:10.1111/j.2517-6161.1995.tb02031.x

© 2025 Koc D et al. *JAMA Network Open.*
