# Supplementary material for: Perinatal Maternal Depressive Symptoms and Brain Connectivity Among 9- to 15-Year-Old Offspring
Source: JAMA Netw Open. 2025 Jul 31;8(7):e2523978. doi: 10.1001/jamanetworkopen.2025.23978 (PMC12314728; doi:10.1001/jamanetworkopen.2025.23978)
Supplement: Supplement 2. — Data Sharing Statement [file jamanetwopen-e2523978-s002.pdf]

Data Sharing Statement

Koc. Perinatal Maternal Depressive Symptoms and Brain Connectivity Among 9- to 15-Year-Old Offspring. *JAMA Netw Open*. Published July 31, 2025.
doi:10.1001/jamanetworkopen.2025.23978

Data
Data available: No

Additional Information
Explanation for why data not available: Access to data from the Generation R Study is subject to strict procedures to ensure compliance with the European Data Protection Regulation, specifically the General Data Protection Regulation (GDPR). Data from the Generation R Study may be available upon request to the study's Management Team (email: datamanagementgenr@erasmusmc.nl). Prospective users must undergo a thorough approval process, which includes detailed application and review steps to ensure that data usage aligns with GDPR requirements and the study's data protection standards.
